# Fetuin-A-Containing Calciprotein Particles Reduce Mineral Stress in the Macrophage

**DOI:** 10.1371/journal.pone.0060904

**Published:** 2013-04-08

**Authors:** Edward R. Smith, Eric Hanssen, Lawrence P. McMahon, Stephen G. Holt

**Affiliations:** 1 Department of Renal Medicine, Eastern Health Clinical School, Monash University, Melbourne, Victoria, Australia; 2 Electron Microscopy Unit, Bio21 Molecular Science and Biotechnology Institute, University of Melbourne, Melbourne, Victoria, Australia; University of California Merced, United States of America

## Abstract

The formation of fetuin-A-containing calciprotein particles (CPP) may facilitate the clearance of calcium phosphate nanocrystals from the extracellular fluid. These crystals may otherwise seed extra-osseous mineralization. Fetuin-A is a partially phosphorylated glycoprotein that plays a critical role in stabilizing these particles, inhibiting crystal growth and aggregation. CPP removal is thought to be predominantly mediated by cells of the reticuloendothelial system via type I and type II class A scavenger receptor (SR-AI/II). Naked calcium phosphate crystals are known to stimulate macrophages and other cell types *in vitro*, but little is known of the effect of CPP on these cells. We report here, for the first time, that CPP induce expression and secretion of tumour necrosis factor (TNF)-α, interleukin (IL)-1β in murine RAW 264.7 macrophages. Importantly, however, CPP induced significantly lower cytokine secretion than hydroxyapatite (HAP) crystals of equivalent size and calcium content. Furthermore, CPP only had a modest effect on macrophage viability and apoptosis, even at very high levels, compared to HAP crystals, which were strongly pro-apoptotic at much lower levels. Fetuin-A phosphorylation was found to modulate the effect of CPP on cytokine secretion and apoptosis, but not uptake via SR-AI/II. Prolonged exposure of macrophages to CPP was found to result in up-regulated expression of SR-AI/II. CPP formation may help protect against some of the pro-inflammatory and harmful effects of calcium phosphate nanocrystals, perhaps representing a natural defense system for calcium mineral stress. However, in pathological states where production exceeds clearance capacity, these particles may still stimulate pro-inflammatory and pro-apoptotic cascades in macrophages, which may be important in the pathogenesis of vascular calcification.

## Introduction

Problems associated with mineral stress are known to occur in a number of pathologies, especially in renal dysfunction. These disorders are associated with the ectopic deposition of crystalline mineral, particularly within the vasculature, mostly in the form of basic calcium phosphate (BCP). Nanocrystals of BCP consisting of calcium hydroxyapatite (Ca_10_(PO_4_)_6_(OH)_2_), or its precursor, octacalcium phosphate (Ca_8_(HPO_4_)_2_(PO_4_)_4_ ⋅5H_2_O), have been reported to strongly stimulate a number of cell types, *in vitro*. The rise in intracellular calcium concentration that accompanies lysosomal dissolution of internalized BCP crystals, or calcium influx following direct or receptor-mediated crystal-cell interactions, induces the synthesis of various intracellular and secreted mediators, and alters membrane permeability and cell viability.

In the macrophage, BCP crystals induce secretion of tumour necrosis factor (TNF)-α, interleukin (IL)-1β and IL-18, via Toll-like receptor 4, protein kinase C-α/mitogen-activated protein kinase and nucleotide-binding domain, leucine-rich-repeat-containing family pyrin domain-containing 3 (NLRP3) inflammasome-dependent pathways [1]–[5]. BCP crystals have also been shown to induce the production of osteogenic proteins by vascular smooth muscle cells (VSMC), and cell death at high levels [6], [7]. In fibroblasts, on the other hand, exposure to BCP results in matrix metalloproteinase production and a proliferative response [8]–[11]. BCP-induced apoptosis of VSMC may lead to atherosclerotic plaque destabilization and rupture, while in the context of medial arteriosclerosis, enhanced VSMC mineralization may potentiate the loss of large vessel compliance [12], [13]. BCP-induced inflammation also contributes to cartilage deterioration, subchondral remodeling and synovitis seen in severe osteoarthritis and other destructive arthopathies [14], [15]. Indeed intra-articular deposition of BCP crystals is strongly associated with the severity of cartilage degeneration [16]. Consequently, BCP crystals are thought to play an important, but cell-specific, role in the genesis of a number of pathologies.

Crystal size appears to be key a determinant of pro-inflammatory and apoptotic potency. Crystals isolated from human aortic and carotid atherosclerotic plaques have been found to range in size from 50 nm to 8 µm [7], [17], however, studies in both the macrophage and VSMC suggest that only crystals less than 1–2 µm in diameter stimulate a strong response [7], [18]. Interestingly, in studies of synthetic hydroxyapatite crystals released from prosthetic implants, Laquerriere *et al* reported that needle-shaped particles were most potently inflammatory [19], thus crystal shape may also be important. To date, many studies have relied on synthetic BCP crystals to assess their biologic effect, however, *in vivo*, ‘naked’ crystals are not found in the circulation and cells are unlikely to be exposed to insoluble mineral alone as it becomes readily coated with various protein components of the extracellular fluid in which they are bathed. Indeed, a study by Terkeltaub and colleagues several decades ago showed that serum-coating of hydroxyapatite significantly inhibited crystal-induced neutrophil stimulation [20]. This inhibitory effect was found to be principally due to the crystal-bound serum protein, fetuin-A. Fetuin-A (or α_2_-Heremans Schmid glycoprotein) is an abundant, partially phosphorylated, glycoprotein secreted by the liver, which plays a critical role in the formation and stabilisation of high molecular weight protein-mineral complexes in extracellular fluid [21]. Fetuin-A binds and sequesters insoluble mineral nuclei, forming soluble colloidal calciprotein particles (CPP), thereby inhibiting crystal growth and aggregation [22]. Recent studies by Herrmann and colleagues have provided compelling evidence that fetuin-A-containing CPP facilitate clearance of mineral nanocrystals by phagocytic cells of the reticuloendothelial system via the type I and type II class A scavenger receptor (SR-AI/II) [23]. Theoretically therefore, prompt clearance of CPP may prevent seeding of extra-osseous calcification and other potentially harmful effects of BCP.

In mice, ablation of the fetuin-A gene results in extensive soft-tissue and myocardial calcification [24]–[26], while in humans with Chronic Kidney Disease (CKD), fetuin-A deficiency has been associated with increased arterial calcification scores and higher mortality rates [27], [28]. In a cohort of CKD patients, Hamano *et al* found that serum fetuin-A-containing CPP levels were strongly correlated with CT coronary artery calcification scores [29]. Expanding on these findings, we reported that higher CPP levels were independently associated with aortic stiffness and serum inflammatory markers, in a well-described cohort of pre-dialysis CKD patients [30]. Subsequently, we have found measurable CPP levels, normally undetectable in healthy controls, in inflamed patients with chronic rheumatological disease, but without renal impairment [31]. Intriguingly, and consistent with an earlier study by Matsui *et al* on a rat model of CKD [32], we have also found that virtually all of the fetuin-A circulating in CPP was in the phosphorylated state [30]. The functional significance of this remains obscure however, as fetuin-A phosphorylation does not appear to be a requisite for CPP formation and inhibitory activity in solution [30], [33].

Given the apparent strong association between CPP levels and inflammatory status, and the known pro-inflammatory response of macrophages to calcium phosphate nanocrystals, the main aim of the present study was to compare the effect of fetuin-A-containing CPP and synthetic hydroxyapatite (HAP) crystals on the inflammatory response, and viability, of murine RAW 264.7 macrophages *in vitro*. Further to this, we investigated the effect of fetuin-A phosphorylation on CPP uptake, and cell fate, and the effect of CPP exposure on SR-AI/II expression.

## Materials and Methods

### Chemicals and Reagents

All chemicals were purchased from Merck Millipore unless otherwise stated. Synthetic hydroxyapatite crystals (<200 nm in diameter) were obtained from Sigma-Aldrich (# 702153). High-purity human serum albumin was sourced from Calbiochem (#126658).

### Preparation of Proteins and Calciprotein Particles

Human fetuin-A was obtained from Calbiochem (Cat# 362199) and purified by size exclusion chromatography using a HiPrep™ 16/60 Sephacryl S-100 HR column (GE Healthcare Life Sciences) equilibrated in Tris-buffered saline (TBS), and monitored at λ_280_. Dephosphorylated fetuin-A (dpFet-A) was produced by enzymatic hydrolysis of purified natively-phosphorylated fetuin-A (npFet-A) with high activity bovine intestinal alkaline phosphatase (ALP) in 0.10 M Tris-HCl pH 9.0 containing 0.05 M MgCl_2_, 0.1 mM dithiothreitol for 12 h at 37°C, as previously described [30]. Dephosphorylation was monitored by phosphate-affinity SDS-PAGE using a polyacrylamide-bound Mn^2+^-phosphate-binding tag (Phos-tag, Nard Institute, Hyogo, Japan), also as previously described [30]. dpFet-A was exchanged into TBS and purified from the reaction mix using a centrifugal ultrafiltration unit with a 7 kDa MWCO membrane (Thermo Scientific), and by gel filtration using a HiPrep™ 16/60 Sephacryl S-200 HR column (GE Healthcare Life Sciences) equilibrated in TBS. Relevant fractions were pooled and concentrated by ultrafiltration using a centrifugal unit with a 10 kDa MWCO membrane (Sartorius AG). Purity was assessed by SDS-PAGE and Western blotting with polyclonal goat anti-human fetuin-A antibody (#RD184036100, Biovendor). Final protein concentration was determined by bicinchonic acid (BCA) assay at 562 nm (Thermo Scientific). npFet-A and dpFet-A fractions were labeled with AlexaFluor 488 carboxylic acid TFP ester using a commercially available kit (Life Technologies), separated from free label using a Zebra desalt centrifugal ultrafiltration unit with a 7 kDa MWCO membrane (Thermo Scientific), snap-frozen in liquid N_2_ and stored at −80°C until use.

Synthetic fetuin-A-containing CPP were prepared as previously described [22]. Briefly, cryoprecipitates and aggregates were cleared from protein stock solutions by centrifugation for 15 min at 12000×g and 4°C. 0.22 µm-filtered precipitation mix containing 1 mg/mL fetuin-A, 10 mmol/L CaCl_2_, 6 mmol/L Na_2_HPO_4_, 140 mmol/L NaCl, 50 mmol/L Tris-HCl (pH 7.4) was incubated for 24 h at 37°C. CPP were harvested using centrifugal filter units with a 300 kDa MWCO membrane (Sartorius AG). Total protein concentration was determined by BCA assay, fetuin-A by ELISA (Biovendor) and the ionic calcium content was measured by graphite furnace atomic absorption spectrometry after acidification in 2 mol/L HCl for 24 h to allow full crystal dissolution.

CPP were also purified from the pooled serum (30 mL) of 10 patients with pre-dialysis CKD. The CPP-containing fraction was pelleted by centrifugation for 2 h at 22000×*g* and 4°C, washed 3 times with ice-cold TBS before being re-suspended in warmed buffer prior to separation by affinity chromatography using an anti-human fetuin-A IgG (Biovendor) coupled CNBr-activated Sepharose 6 MB resin (GE Healthcare Life Sciences). Fetuin-A-containing fractions were identified by Western blotting with anti-human fetuin-A IgG (Biovendor), pooled and concentrated by ultrafiltration with 300 kDa MWCO filter units. Total protein, fetuin-A and calcium content were determined as before (79 µg/mL protein, 33 µg/mL fetuin-A, 15 µg/mL calcium). Participating patients gave written informed consent. The study was approved by local regional ethics committee (Eastern Health Research and Ethics Committee ref: LLR31/1112) and was conducted in accordance with the Declaration of Helsinki.

### Transmission Electron Microscopy and X-ray Elemental Microanalysis

For cryo-electron microscopy, the sample was plunged frozen in liquid ethane before observation on a Tecnai F30 (FEI, the Netherlands) operating at 300 kV. Each micrograph represents an exposure of 2,000 electrons per nm. For cell observation, isolated cells were fixed at 4°C in 0.1 M sodium cacodylate pH 7.4 containing 5 mM calcium chloride, 1% glutaraldehyde and 1.5% formaldehyde. Cells were post-fixed in 2% osmium tetroxide and serially dehydrated before embedding in Epon. Seventy nanometer sections were observed with a Tecnai F30 and micrograph acquired with a 2 k×2 k Ultrascan camera (Gatan, CA, USA). For energy dispersive spectroscopy, isolated particles were absorbed on a carbon coated copper grid for 30 seconds, rinsed with distilled water and air-dried. The measurements were made in STEM mode on the Tecnai F20 equipped with an EDAX detector (NJ, USA) with an ultra-thin window.

Immunogold labeling was performed using a goat anti-human fetuin-A antibody (1∶100 dilution) and 10 nm gold-conjugated rabbit anti-goat secondary antibody (1∶20 dilution) from Aurion (#810.077). Goat anti-human ALP antibody (#sc-15065, Santa Cruz Biotechnology) was substituted for the anti-fetuin-A primary antibody as a control.

### RAW 264.7 Cell Culture and Treatment

Mouse monocyte macrophage RAW 264.7 cells were obtained from ECACC (Sigma) and were used between passages 3 and 11. Cells were seeded in 24-well plates at a density of 25000 cells per well and left to attach for 16 h in DMEM culture medium supplemented with 10% FBS, 2 mmol/L L-glutamine, 100 IU/mL penicillin, 100 µg/mL streptomycin (all Gibco), at 37°C in a humidified atmosphere with 5% CO_2_. All experiments were performed in the presence of 10% FBS unless otherwise stated. HAP crystals (Sigma-Aldrich) were spin-filtered using cartridges with a 300 kDa MWCO membrane (Sartorius AG) and exchanged into TBS before addition to stock culture media. Nanoparticle preparations were sonicated in culture medium for 1 min before use and were endotoxin free (<0.1 EU/mL). Cells were then incubated with CPP or HAP-containing culture medium or media control, at the concentrations indicated, for 24, 48 or 72 h. Since one of the primary objectives of this study was to compare the effects of CPP and HAP, we defined ‘particle exposure’ in terms of crystal calcium content. Concentration ranges were chosen to reflect potentially pathological CPP-associated calcium levels. In serum samples from CKD patients we previously measured CPP-associated calcium levels in the range 0.3–10 µg/mL (equivalent to approx. 0.5–16 µg/mL HAP) [30]. However, given that CPP may accumulate to much higher levels at sites of mineral stress (e.g. atherosclerotic lesions) we determined to investigate that effect of nanoparticles over a broader concentration range: 10–100 µg/mL (equivalent to approx. 16–160 µg/mL or 6–60 µg/m^2^ HAP).

### CPP Binding/uptake Experiments

For confocal microscopy RAW 264.7 cells were seeded in 2-well coverslips (Invitrogen) at a density of 10000 cells per well for 24 h in DMEM culture medium supplemented with 10% FBS, 2 mmol/L L-glutamine, 100 IU/mL penicillin and 100 µg/mL streptomycin. Adherent cells were then incubated with AlexaFluor488-labelled fetuin-A-containing synthetic CPP in complete culture medium for 30 minutes at 37°C. In order to differentiate between cell-bound and internalized CPP, rabbit anti-AlexaFluor488 IgG was used to quench cell-surface fluorescence. At given time points, cells were washed with ice-cold PBS and incubated in PBS as control, or 80 µg/mL anti-AlexaFluor488 IgG in PBS (#A-11094, Invitrogen) for 1 h at 4°C. Cells were then fixed in 4% paraformaldehyde (PFA) in PBS for 15 min at RT and mounted in ProLong Gold Antifade Reagent (Invitrogen). Images were captured using a Fluoview FV1000-IX81 confocal microscope (Olympus).

To study CPP internalisation kinetics, RAW 264.7 cells were seeded on 96-well plates at a density of 5000 cells per well in DMEM culture medium supplemented with 10% FBS, 2 mmol/L L-glutamine for 12 h at 37°C. Adherent cells were then incubated with AlexaFluor488-labelled fetuin-A-containing synthetic CPP (100 µg/mL), and then subsequently with quenching antibody or PBS alone as described above. The efficiency of cell-surface quenching with anti-AlexaFluor488 IgG was estimated by measuring the fluorescence after incubation of cells with labeled CPP at 4°C, as CPP uptake should be minimal at this temperature. Unquenchable fluorescence was determined at each time point and accounted for 5 to 15% of the total cell-associated signal. Internalization of CPP was calculated using the ratio of quenched signal (intracellular CPP) to unquenched signal (combined cell-surface and intracellular CPP) after correction for the unquenchable fluorescence as described by Göstring *et al* [34]. Total cell-associated fluorescence was measured using a Synergy HT multimode plate reader with appropriate excitation/emission filters (BioTek).

Given that FBS contains large amounts of bovine fetuin, serum-free medium (Opti-MEM I, Gibco) was used to culture cells for the assessment of monomeric fetuin-A uptake. Cells were washed and cultured in serum-free medium for 2 h prior to treatment with CPP or HAP-containing medium containing purified human fetuin-A at the concentrations indicated.

To block apoptosis, cells were pre-incubated with 20 µmol/L z-VAD-fmk (Calbiochem) for 1 h before treatment with CPP/HAP. To block endocytosis via SR-AI, cells were pre-incubated with rat monoclonal anti-mouse SR-AI blocking antibody (10 µg/mL, #HM1061, Hycult Biotech) or rat IgG_2b_ isotype control (10 µg/mL, #14–4031, eBioscience) for 1 h prior to addition of CPP or HAP-containing culture medium for a further 24 h.

### Cell Viability, Proliferation and Apoptosis Assays

Cell viability was assessed by MMT assay of mitochondrial activity (Sigma). RAW 264.7 cells were plated in 96-well plates at 5000 cells per well and left overnight to attach in DMEM culture medium supplemented with 10% FBS, 2 mmol/L L-glutamine, 100 IU/mL penicillin and 100 µg/mL streptomycin. After 16 h, cells were switched to culture medium containing CPP or HAP crystals, at the stated concentrations, and incubated for 24 h. MMT solution (5 mg/ml) was then added (10% vol/vol) and the cells incubated at 37°C for a further 3 h. Formazan crystals formed by living cells were dissolved in MMT solvent (0.1 mol/L HCl in anhydrous isopropanol), and the absorbance measured at 570 nm (650 nm background subtracted).

For assessment of macrophage proliferation, RAW cells were plated in 24-well plates at 10000 cells per well and left overnight in culture medium. After attachment, the medium was changed to DMEM supplemented with only 1.0% FBS for 48 h to induce quiescence. This medium was then replaced with growth medium (DMEM supplemented with 10% FBS, 2 mmol/L L-glutamine) with or without CPP/HAP, at the stated concentrations, for 48 h. To quantitate the number of cells in culture, the medium was aspirated and the cells gently scraped. Viable cells were identified by trypan blue exclusion and were counted in a hemocytometer.

For the detection of apoptosis, cells were plated in 96-well plates as for MMT assay, and then incubated in culture medium containing CPP/HAP at the stated concentrations for 24 h (each treatment, n = 16 replicates). After fixation (4% PFA in PBS for 15 min at RT) and permeabilisation of the cells (0.25% Triton X-100 in PBS), plates were processed for a TUNEL (fluorescence transferase-mediated dUTP nick-end labeling) based assay using the Click-iT TUNEL Alexa Fluor 488 Imaging Assay (Invitrogen). DNase I-treated cells were used as a control. To confirm the findings by TUNEL assay, replica plates were fixed with 80% methanol in PBS and then processed for assay using the ssDNA Apoptosis ELISA kit from Chemicon International. This assay has high sensitivity (>500 apoptotic cells) and high specificity for apoptosis showing no signal for hyperthermia/detergent-induced necrosis or DNA breaks induced by hydrogen peroxide. S1 nuclease-treated cells were used as a negative control (Invitrogen). Caspase-3 activity of whole-cell lysates was assessed using the EnzChek kit obtained from Molecular Probes (Invitrogen). For this assay, cells were seeded at a density of 25000 per well in 24-well plates and then treated with CPP/HAP-containing culture medium as before. Lysates were also prepared as previously described. Activity was detected by cleavage of the fluorogenic substrate Z-DEVD-AMC and the signal measured in a fluorescence microplate reader. Caspase 3 inhibitor peptide, Ac-DEVD-CHO, was added to selected samples to ensure specificity of the signal.

### ELISA

Supernatants were collected, cleared from particulate debris by centrifugation for 10 min at 1000×*g* and 4°C, and stored at −80°C until analysis. TNF-α and IL-1β concentrations were determined using kits from R&D Systems. 8-iso-PGF_2α_ was measured in whole cell lysates using a kit from Cayman Chemical and normalized to total protein concentration determined by BCA assay (Thermo Scientific). All measurements were made in duplicate and the assays performed according to the manufactures instructions.

### Western Blotting

Cells were lysed in ice-cold lysis buffer containing 50 mmol/L Tris-HCl, pH 8.0, 5 mmol/L EDTA, 150 mmol/L NaCl, 1% Triton X-100 and protease inhibitor cocktail (Sigma) for 30 min. Lysates were stored at −80°C until analysis. Total protein concentration was determined by BCA assay (Thermo Scientific). Proteins (5 µg/lane) were separated on 4–12% NuPAGE Bis-Tris pre-cast gels (Invitrogen) and transferred onto PVDF using the iBlot dry-blotting system (Invitrogen). Membranes were blocked in 5% non-fat milk for 2 h at RT before being probed with specific goat antisera. Murine SR-AI was detected using a polyclonal anti-SR-AI antibody (1∶250 dilution) from Santa Cruz Biotechnology (# sc-20444) and normalized to β-actin levels detected with polyclonal anti-β-actin antibody (1∶250 dilution) (Santa Cruz Biotechnology). Signals were visualised with rabbit HRP-conjugated anti-goat IgG (1∶10000 dilution) (eBioscience, San Diego, CA, USA) and the Immu-Star chemiluminescence Western C kit (Bio-Rad) on a VersaDoc 4000 MP imaging platform (Bio-Rad). Relative band intensity was determined using Image Lab software (Bio-Rad).

### Expression Analysis

After treatment with the indicated reagents, cells were washed once with ice-cold PBS to remove unbound particles before total RNA was extracted using the Bio-Rad AURUM total RNA mini kit. Reverse transcription (∼1 µg per extract) was performed using iScript RT supermix (Bio-Rad). cDNA products were diluted 1∶5 in DNAse-free water prior to storage at −20°C until quantitative PCR analysis. Real-time PCR analysis using SsoAdvanced SYBR Green Supermix (Bio-Rad), and pre-designed, and pre-optimized qSTAR qPCR primer pairs (Origene) was performed on a CFX96 Connect RT-PCR detection system with CFX Manager software version 3.0 and Precision melt analysis version 1.2 (Bio-Rad). Primer sequences are listed in Table 1. Reactions mixtures included 250 nM of each primer, 100 ng cDNA template, 10 µL supermix, made up to a total volume of 20 µL with DNase/RNase-free water. Thermal cycler conditions were 95°C for 30 s, then 40 cycles of 95°C for 10 s, 60°C for 30 s followed by melt curve analysis from 60–95°C in 0.5°C increments. PCR amplification specificities were evaluated by the examination of melt curves to confirm the presence of single gene peaks. Dilution experiments were performed to ensure reaction efficiencies of each primer pair were comparable. All results were normalized to B2 M as recommended by Stephens *et al* [35], and expressed as the ratio of target mRNA levels (relative to B2 M) in treated vs. control cells using the 2-^ΔΔCt^ method.

**Table 1:** PCR primer sequences.

Gene target	Accession No.	Forward primer (5′–3′)	Reverse primer (5′–3′)
B2 m	NM_009735	ACAGTTCCACCCGCCTCACATT	TAGAAAGACCAGTCCTTGCTGAAG
Tnfa	NM_013693	GGTGCCTATGTCTCAGCCTCTT	GCCATAGAACTGATGAGAGGGAG
Il-1b	NM_008361	TGGACCTTCCAGGATGAGGACA	GTTCATCTCGGAGCCTGTAGTG
Casp-3	NM_009810	GGAGTCTGACTGGAAAGCCGAA	CTTCTGGCAAGCCATCTCCTCA
SR-AI/II	NM_001113326	CGCACGTTCAATGACAGCATCC	GCAAACACAAGGAGGTAGAGAGC

Abbreviations: B2 m, β_2_-microglobuin; Tnfa, tumour necrosis factor-α; Il, interleukin; Casp, capsase; SR-A, class A scavenger receptor.

### Statistical Analysis

All experiments were performed in triplicate for each treatment and repeated in a minimum of 3 independent experiments. Data are expressed as mean ± SD unless otherwise stated. Unpaired Student’s *t* tests were used to compare two groups and one-way ANOVA with Tukey post-test was performed for the comparison of three or more groups. *P* values <0.05 were considered significant. Analysis was performed using GraphPad Prism version 5.04.

## Results

### Uraemic Human Serum Contains a Heterogeneous Population of Fetuin-A Containing-CPP with an Apatite Polycrystalline Core

Previous reports suggest that fetuin-A-containing CPP undergo spontaneous transformation from relatively small (<100 nm) spherical particles of amorphous calcium phosphate (“primary CPP”), to larger, elongate spindle-shaped particles containing crystalline calcium apatite (“secondary CPP”) [36]. To date, it has not been demonstrated which species are present in uraemic human serum.

**Figure 1:**
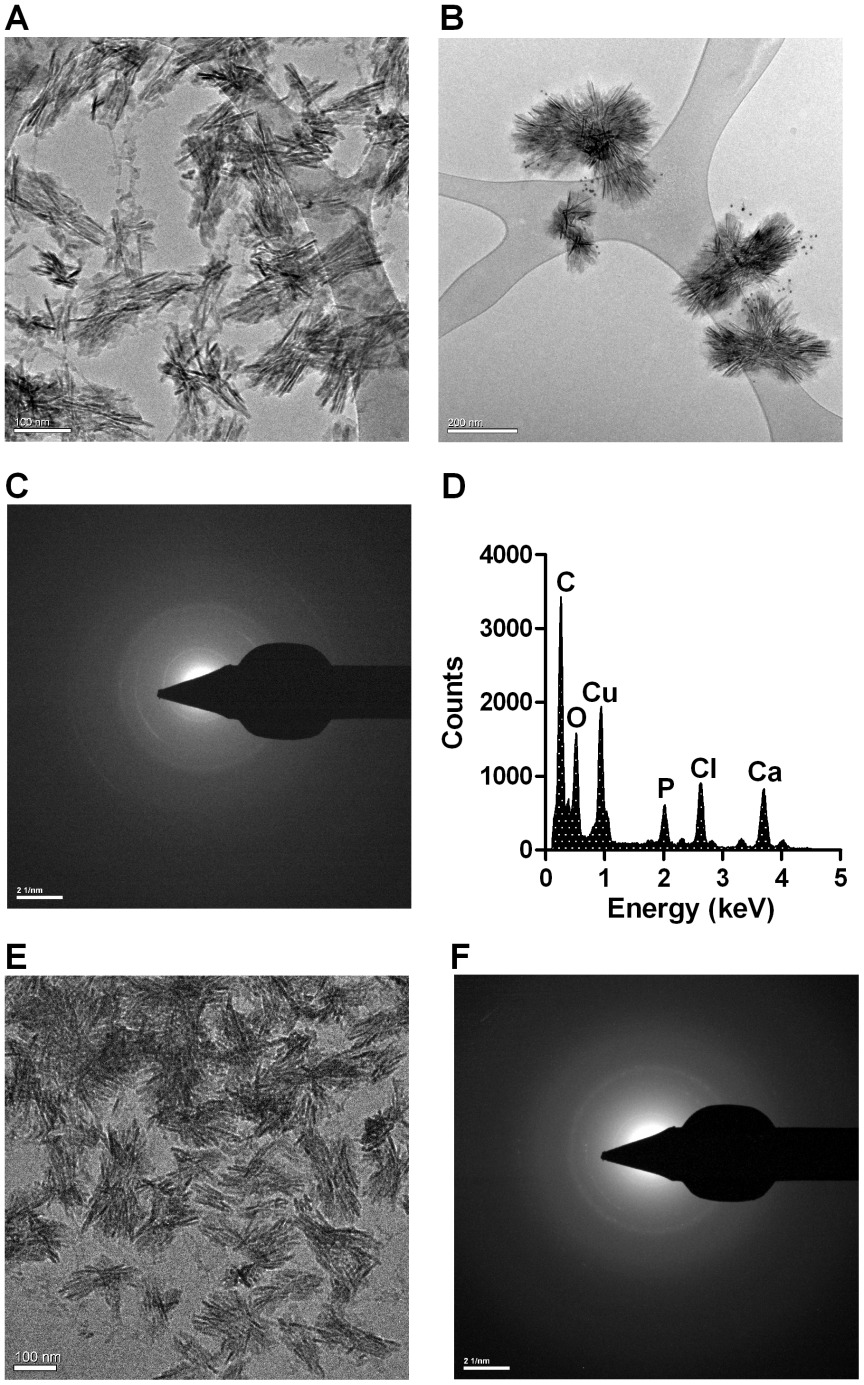
TEM analysis of serum-derived and synthetic CPP. **A** , cryo-TEM image of CPP isolated from uraemic human serum showing a heterogeneous population of elongate, spindle-shaped particles, with an electron-dense core, ranging in size from approximately 80 to 250 nm (bar = 100 nm). **B**, immunogold labeling of fetuin-A showing irregular, but dense decoration of gold (10 nm) at particle surface, consistent with a fetuin-A containing proteinacious shell (bar = 200 nm). **C**, electron diffraction pattern from a typical CPP comprising a series of concentric rings, consistent with a polycrystalline material. **D**, electron dispersive x-ray spectroscopy (EDX) of CPP showing strong peaks for O, P and Ca giving a Ca/P^−^ of 1.67±0.03, consistent with calcium apatite-like material. **E**, cryo-TEM image of synthetic CPP isolated from precipitation mix after 24 h incubation at 37°C, showing similar morphology to serum-derived CPP (bar = 100 nm for E). **F**, electron diffraction analysis of a synthetic CPP showing a similar pattern of concentric rings to serum-derived particle.

Fetuin-A-containing CPP were isolated from pooled uraemic serum by ultracentrifugation and affinity chromatography. Cryo-TEM of this fraction showed the presence of a heterogeneous population of elongate, spindle-shaped particles, with an electron-dense core and ranging in size from approximately 80 to 200 nm (Fig. 1A). The morphology of these particles is most in keeping with that of secondary CPP first described by Jahnen-Dechent and colleagues [37]. Anti-fetuin-A immunogold labeling displayed an irregular but dense decoration of gold at the surface of these particles, consistent with a fetuin-A-containing proteinacious ‘shell’ (Fig. 1B). Electron diffraction patterns of particles yielded a series of concentric rings (Fig. 1C), confirming the presence of material in a polycrystalline form. Energy-dispersive X-ray (EDX) spectral analysis showed strong peaks for Ca, P and O, with a calcium-to-phosphate molar ratio (Ca/P) of 1.67±0.03, consistent with calcium apatite-like material (Fig. 1D).

Given the limited availability of serum-derived CPP material, and the potential for co-purification of bioactive contaminants, subsequent experiments were performed using synthetic secondary CPP, unless otherwise indicated. This also allowed us to specifically evaluate the effect of fetuin-A on crystal handling. Synthetic CPP, isolated from precipitation medium 24 h after mixing, showed near-identical morphology, size and a similar diffraction pattern to serum-derived particles (Fig. 1E and 1F). As expected, EDX analysis showed strong peaks for Ca, P and O, with a Ca/P of 1.67±0.02. Synthetic CPP were found to remain intact and stable in culture medium for at least 5 days at 37°C, with ionic calcium and phosphate concentrations remaining unchanged over this period.

### Pro-inflammatory Cytokine Expression is Attenuated by Fetuin-A-containing CPP Compared to Naked Hydroxyapatite Crystals

**Figure 2:**
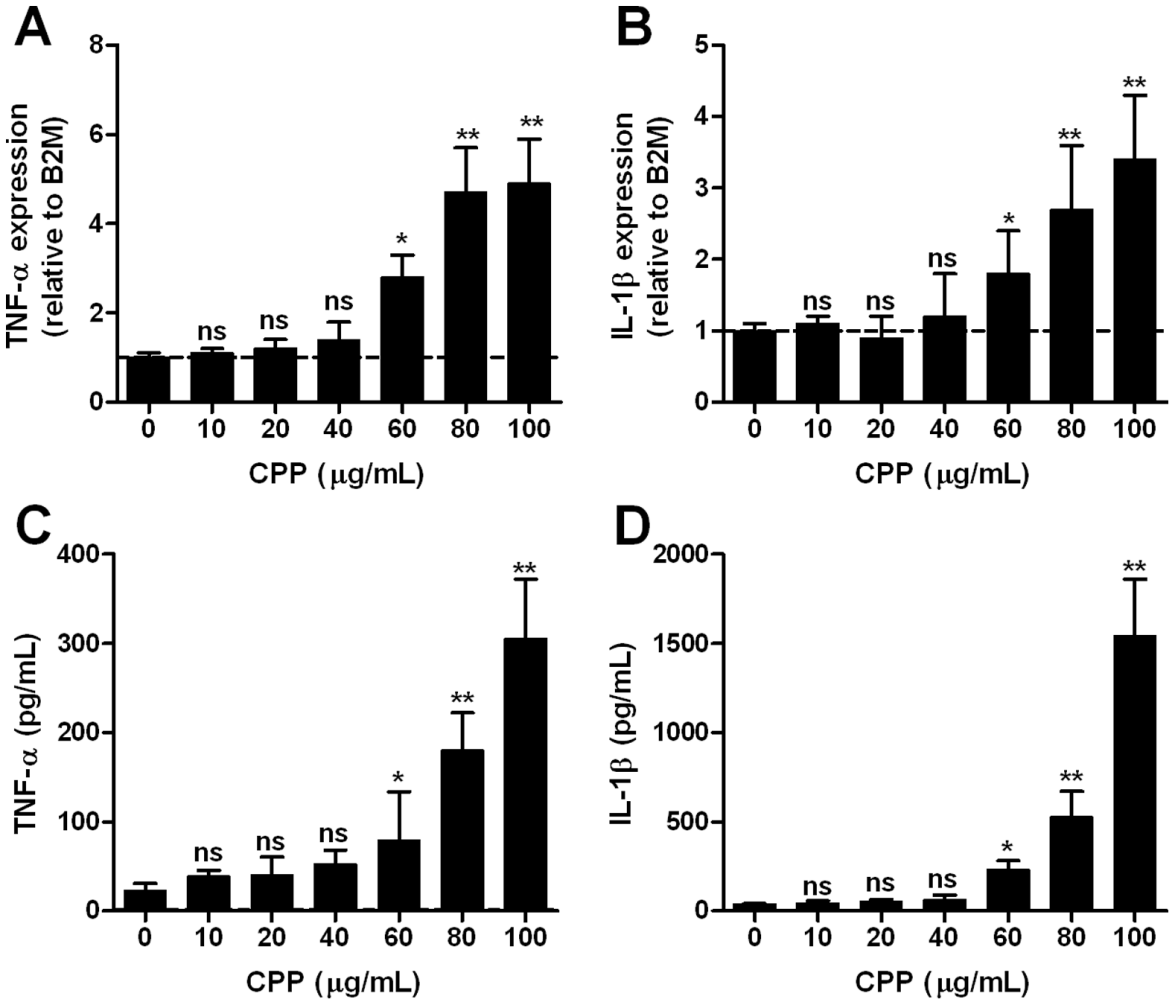
CPP induce TNF-α and IL-1β expression and secretion from macrophages at high levels. Murine RAW 264.7 cells were treated with synthetic CPP-containing culture medium for 24 h at the concentrations indicated. CPP exposure has a dose-dependent effect on **A**, TNF-α and **B**, IL-1β gene expression by qPCR (P for trend both <0.01, ANOVA). mRNA levels were normalized to B2 M and expressed relative to control medium (1: dashed line). Panels **C** and **D**, show the dose-dependent effect of synthetic CPP exposure on TNF-α and IL-1β secretion, respectively (P for trend both <0.01, ANOVA). TNF-α and IL-1β released into culture supernatants were analyzed by ELISA. Determinations were made in quadruplicate in 4 independent experiments and are expressed as mean ± SD. Pairwise comparisons were made using the unpaired *t*-test; ns, not significant (P>0.05); *P<0.05; **P<0.01.

Several studies have reported that BCP nanocrystals (mainly as hydroxyapatite, HAP) strongly stimulate the secretion of TNF-α and IL-1β by monocyte-macrophage [1]–[5]. First, we tested whether CPP induce a similar response in RAW 264.7 macrophages. Treatment of macrophages with CPP for 24 h (final concentration: 10–100 µg/mL) resulted in a dose-dependent increase in the expression of TNF-α and IL-1β, however this effect only became significant at CPP levels above 80 µg/mL (Fig. 2A and 2B, respectively). Up-regulated cytokine expression was found to persist at 48 and 72 h post-treatment with 80 µg/mL CPP (data not shown). Consistent with the increase in gene expression, protein levels of TNF-α and IL-1β in the culture medium also increased significantly with higher CPP levels, but likewise only became significantly elevated at CPP levels above 60 µg/mL (Fig. 2C and 2D, respectively).

**Figure 3:**
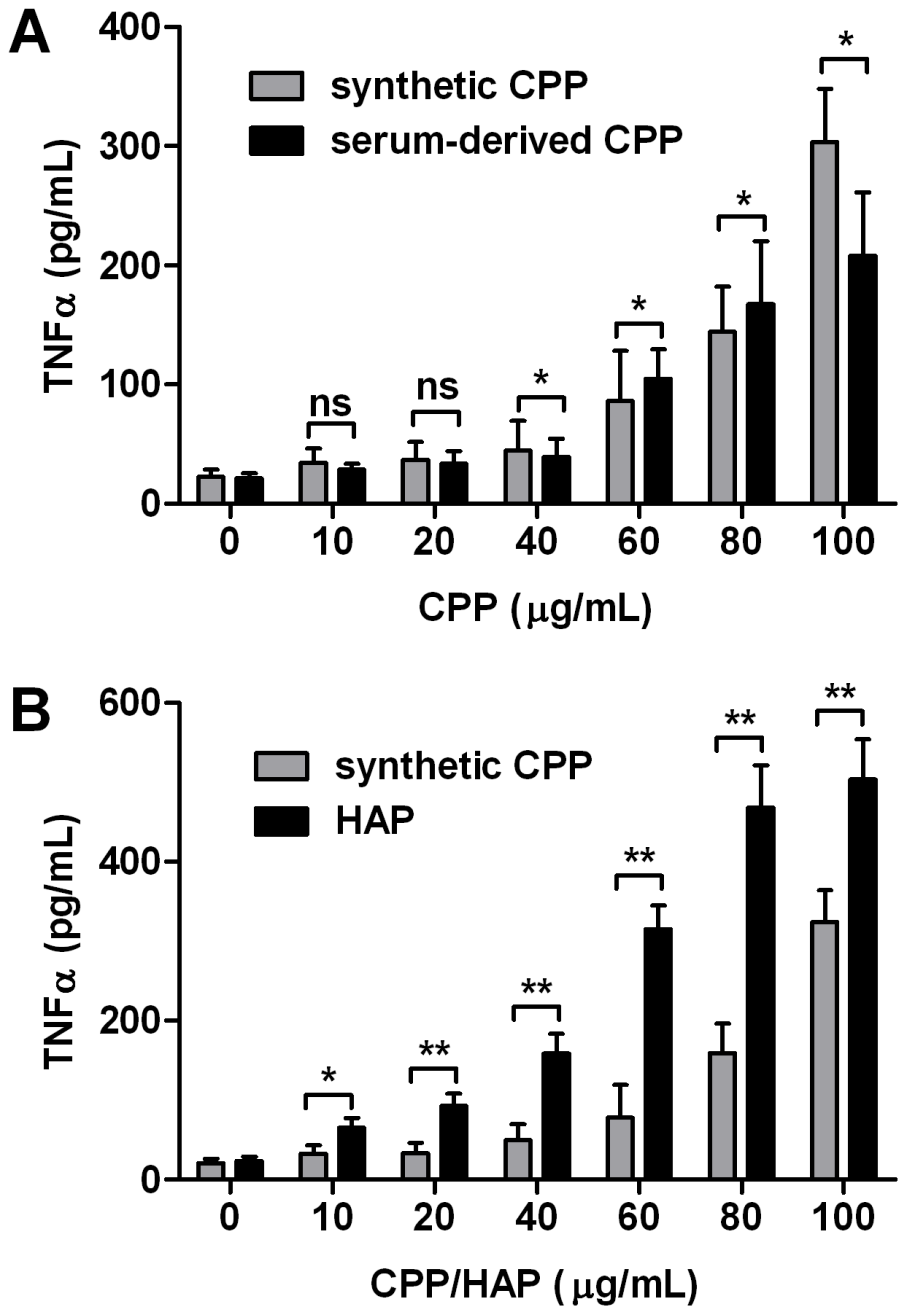
Serum-derived CPP induce less TNF-α secretion from macrophages than synthetic CPP or naked hydroxyapatite crystals. Murine RAW 264.7 cells were treated with synthetic CPP, serum-derived CPP or hydroxyapatite (HAP)-containing culture medium for 24 h at the concentrations indicated. **A**, Comparison of the effects of synthetic (grey) and serum-derived (black) CPP on TNF-α secretion by macrophages (P for trend, both <0.01) **B**, Comparison of the effects of synthetic CPP (grey) and HAP nanocrystals (black) of equivalent size (100–200 nm) and calcium content on TNF-α secretion by macrophages. TNF-α released into culture supernatants was analyzed by ELISA (P for trend, both <0.01). Determinations were made in quadruplicate, in 3 independent experiments and are expressed as mean ± SD. Pairwise comparisons were made using the unpaired *t*-test; ns, not significant (P>0.05); *P<0.05; **P<0.01.

Comparison of cells treated with either synthetic or serum-derived CPP showed a parallel trend towards increased TNF-α expression with increasing CPP levels (Fig. 3A). Serum-derived CPP, however, elicited significantly lower levels of TNF-α expression over synthetic CPP at 100 µg/mL. We next sought to compare the effect of CPP and HAP of equivalent size (100–200 nm) and calcium content (final concentration: 10–100 µg/mL) on cytokine production after 24 h treatment in respective test media (Fig. 3B). Critically, compared to CPP-treated cells, HAP-treated cells consistently yielded higher cytokine concentrations in the culture medium across the concentration range tested, particularly above 60 µg/mL.

### CPP Induce Oxidative Stress and Apoptosis in RAW 264.7 Cells

**Figure 4:**
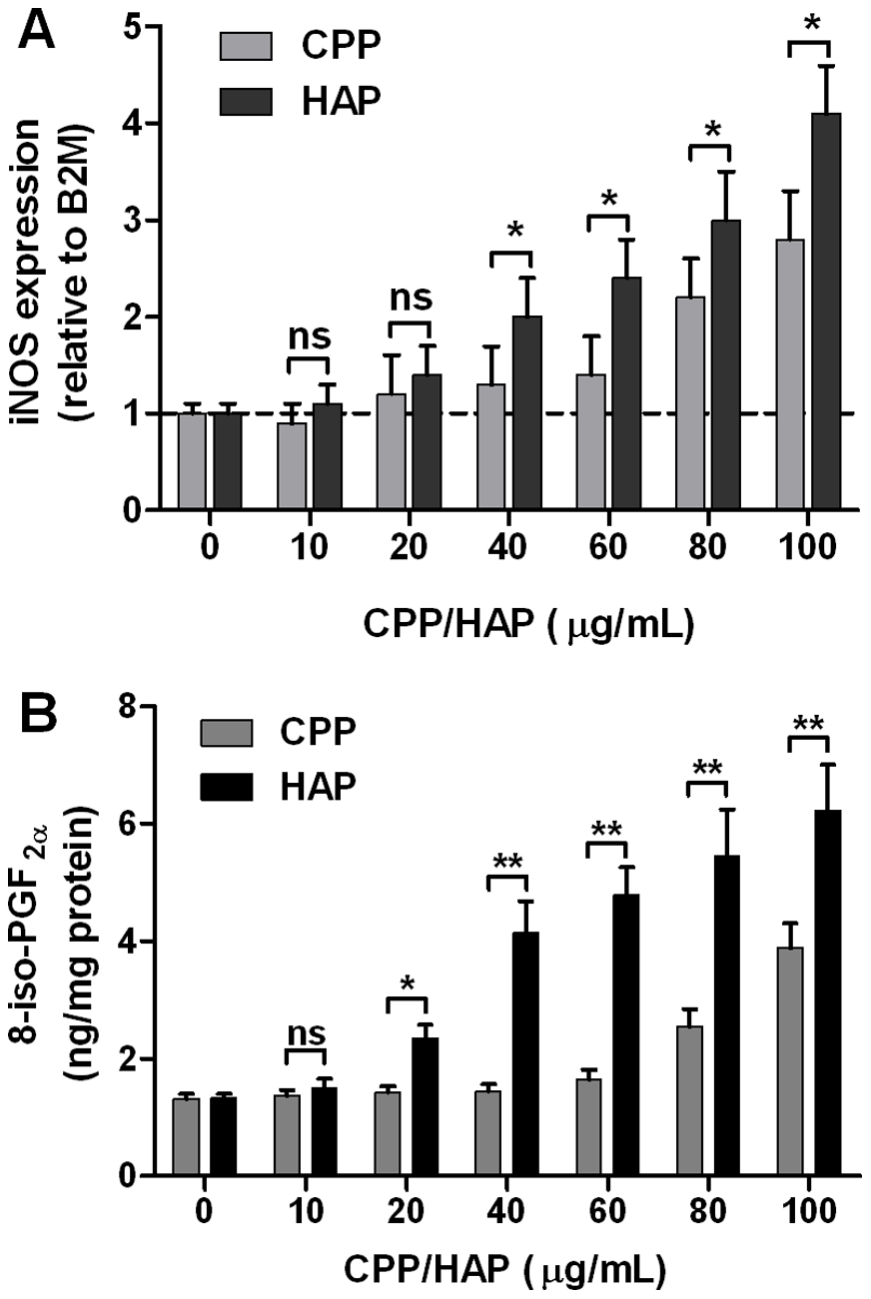
CPP and hydroxyapatite crystals induce iNOS expression and intracellular 8-iso-PGF_2α_ production in macrophage. Murine RAW 264.7 cells were treated with CPP- or hydroxyapatite (HAP)-containing culture medium for 24 h at the concentrations indicated. **A**, shows the dose-dependent effect of CPP or HAP exposure on iNOS gene expression by qPCR (P for trend = 0.01, ANOVA). mRNA levels were normalized to B2 M and expressed relative to control medium (1: dashed line). **B**, shows the dose-dependent effect of CPP or HAP exposure on intracellular 8-iso-PGF_2α_ production (P for trend both<0.01, ANOVA). Whole cell lysates were analyzed by ELISA and expressed as a ratio to total protein (BCA assay). Determinations were made in triplicate, in 3 independent experiments and are expressed as mean ± SD. Pairwise comparisons were made using the unpaired *t*-test; ns, not significant (P>0.05); *P<0.05; **P<0.01.

Previous work, on several cell types, suggests that uptake of BCP crystals also induces the generation of reactive oxygen species (ROS) [2], nitric oxide (NO) [38], cyclooxygenase [15], and inconsistently [2], [39] cause cell death [4], [7], [40]–[42]. In our hands, treatment of RAW 264.7 cells with CPP resulted in a dose-dependent increase in expression of inducible nitric oxide synthase (iNOS), which was accompanied by increased intracellular 8-iso-PGF_2α_ production, a marker of oxidative stress, both of which increased at CPP levels above 80 µg/mL, compared to control levels (Fig. 4A and 4B). In HAP-treated cells however, both iNOS expression and 8-iso-PGF_2α_ levels were markedly elevated at 40 µg/mL after 24 h treatment (Fig. 4A and 4B).

**Figure 5:**
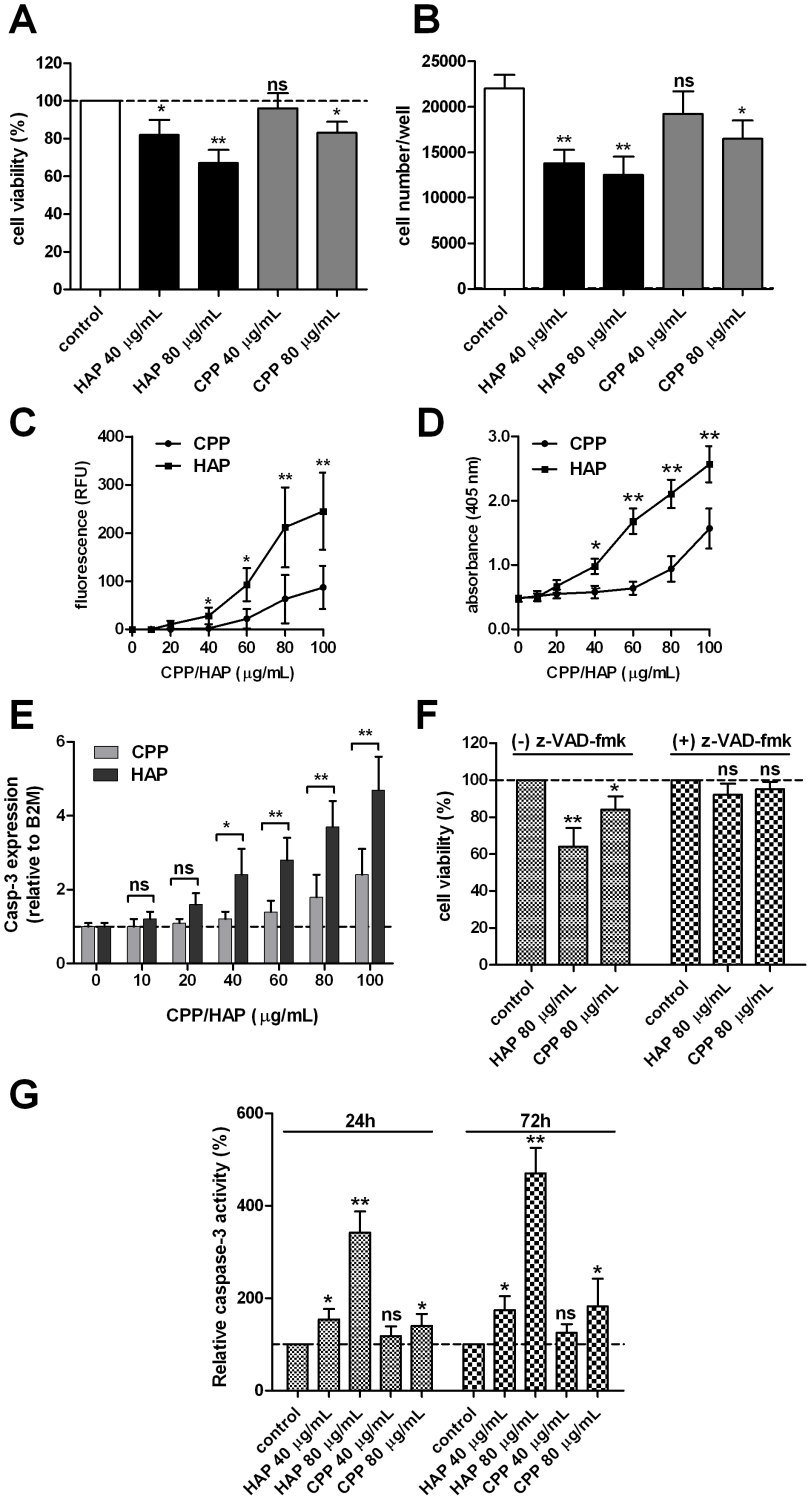
The effect of CPP and hydroxyapatite crystals on macrophage viability and apoptosis. Murine RAW 264.7 cells cultured in hydroxyapatite (HAP)- or CPP-containing medium showed a dose-related reduction in **A**, viability using MTT assay after 24 h compared to cells treated with control medium (100%, dashed line) and **B**, reduction in cell numbers (proliferation) after 48 h incubation (n = 6 for each treatment). Apoptosis was detected in macrophages treated with >40 µg/mL HAP or >80 µg/mL CPP- containing culture medium using **C**, a microplate-based TUNEL assay (n = 8 for each treatment) and **D**, ssDNA levels measured by ELISA (n = 8 for each treatment). **E**, the dose-dependent effect of CPP or HAP exposure on caspase-3 gene expression by qPCR (P for trend both <0.01, ANOVA). mRNA levels were normalized to B2 M and expressed relative to control medium (1: dashed line) (n = 4 for each treatment). **F**, pre-treatment of cells with the pan-caspase inhibitor z-VAD-fmk (20 µmol/L) for 1 h prior to addition of CPP- or HAP-containing culture medium (both 80 µg/mL) for a further 24 h, nullified the effect of either nanocrystal preparation on macrophage viability as assessed by MTT assay (n = 6 for each treatment). **G,** whole cell lysate caspase-3 protease activity was measured by monitoring the cleavage of the synthetic fluorescent substrate, Z-DEVD-AMC and was increased in cells treated with µg/mL HAP or >80 µg/mL CPP- containing culture medium for 24 h and 72 h (n = 4 for each treatment). Determinations were made in 3 independent experiments and are expressed as mean ± SD. Pairwise comparisons were made using the unpaired *t*-test; ns, not significant (P>0.05); *P<0.05; **P<0.01.

Cell viability was assessed by MTT assay at 24 h after treatment with either CPP- or HAP-containing media. While CPP treatment resulted in a significant reduction in macrophage viability at relatively high concentrations (80 µg/mL), HAP-treated cells showed a significant reduction in viability at 40 µg/mL after 24 h (Fig. 5A). To determine whether the reduction in cell viability was due to cell death, synchronized RAW 264.7 cultures were stimulated to proliferate in CPP or HAP-containing media and the number of cells was counted at 72 h post-treatment. Consistent with the apparent loss of viability being due to cell death, the number of cells was significantly reduced after treatment with HAP compared to treatment with CPP-containing medium (Fig. 5B). To investigate whether cells underwent apoptosis during this period we applied a semi-quantitative, microplate-based TUNEL assay to cells after 24 h incubation with CPP or HAP. Again, CPP-treated RAW 264.7 cells showed increased apoptosis at concentrations 80 µg/mL, whereas apoptosis was detected at lower concentrations at 40 µg/mL with HAP-containing medium (Fig. 5C). Comparable findings were made using an immunoassay based on the sensitivity of DNA in apoptotic cells to formamide denaturation. ssDNA levels were detectable above controls in cells treated with HAP at 20 µg/mL, but only at 80 µg/mL with CPP-containing media (Fig. 5D). Expression of caspase-3 was increased after 24 h treatment with 40 µg/mL HAP-containing medium, and at 80 µg/mL with CPP-containing medium (Fig. 5E). Potentially therefore, the high concentrations of pro-inflammatory cytokines observed in culture medium after exposure to high levels of CPP/HAP may be due to release from dead or dying cells. Pre-treatment of cells with z-VAD-fmk (20 µmol/L), a pan-caspase inhibitor, significantly increased cell viability at 24 h compared to cells treated with 80 µg/mL CPP or HAP alone (Fig. 5F). Likewise, cell lysate caspase 3 activities, measured by cleavage of the synthetic fluorescent substrate, Z-DEVD-AMC, were significantly higher in HAP-treated cells compared to CPP-treated cells after incubation for 24 h at >40 µg/mL (Fig. 5G).

### Internalization of CPP by RAW 264.7 Cells

**Figure 6:**
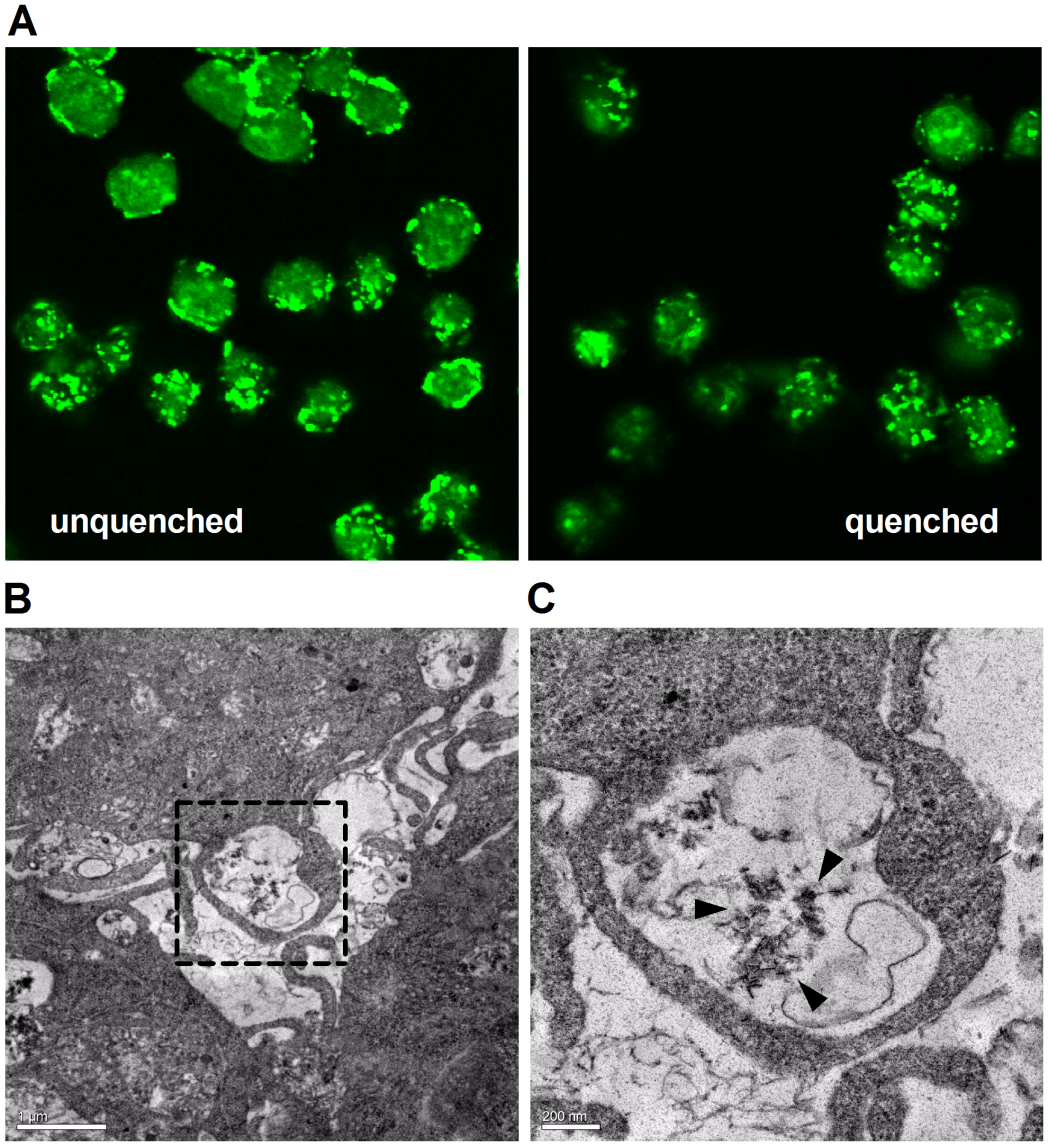
Internalization of CPP by RAW 264.7 cells. **A,** RAW 264.7 macrophage were seeded on coverslips and incubated with 100 µg/mL AlexaFluor488-labeled fetuin-A-containing synthetic CPP (green) in DMEM culture medium supplemented with 10% FBS for 30 minutes at 37°C, with (right-hand side panel) or without (left-hand side panel), quenching of cell-surface fluorescence using anti-AlexaFluor488 IgG (80 µg/mL), or PBS as control, for 1 h at 4°C. Cells were then washed, fixed, mounted in ProLong Gold antifade reagent and visualized by confocal microscopy. **B**, TEM of murine RAW 264.7 macrophage treated with 100 µg/mL CPP-containing culture medium supplemented with 10% FBS for 1 h at 37°C. The dashed box outlines a vacuole containing CPP (bar = 1 µm). **C**, magnified image of box shown in A (bar = 200 nm). Black arrowheads indicate position of vacuolated CPP near the cell surface.

In keeping with the recent report by Herrmann *et al* [23], we found that CPP were rapidly internalized by murine RAW 264.7 cells. Confocal microscopy of cells incubated with AlexaFluor488-labelled fetuin-A-containing CPP (100 µg/mL) in DMEM culture medium containing 10% FBS, showed internalization of particles within 30 minutes (Fig. 6A). Quenching of cell-surface fluorescence with anti-AlexaFluor488 IgG confirmed localization of CPP to intracellular compartments (Fig. 6A, right hand side panel). Consistent with these findings, TEM of RAW 264.7 cells exposed to synthetic (unlabeled) CPP for 1 hour showed uptake of particles into vacuole-like structures near the cell surface (Fig. 6B & 6C). Vacuolar inclusions containing CPP-like particles were not observed in cells incubated in vehicle culture medium alone.

**Figure 7:**
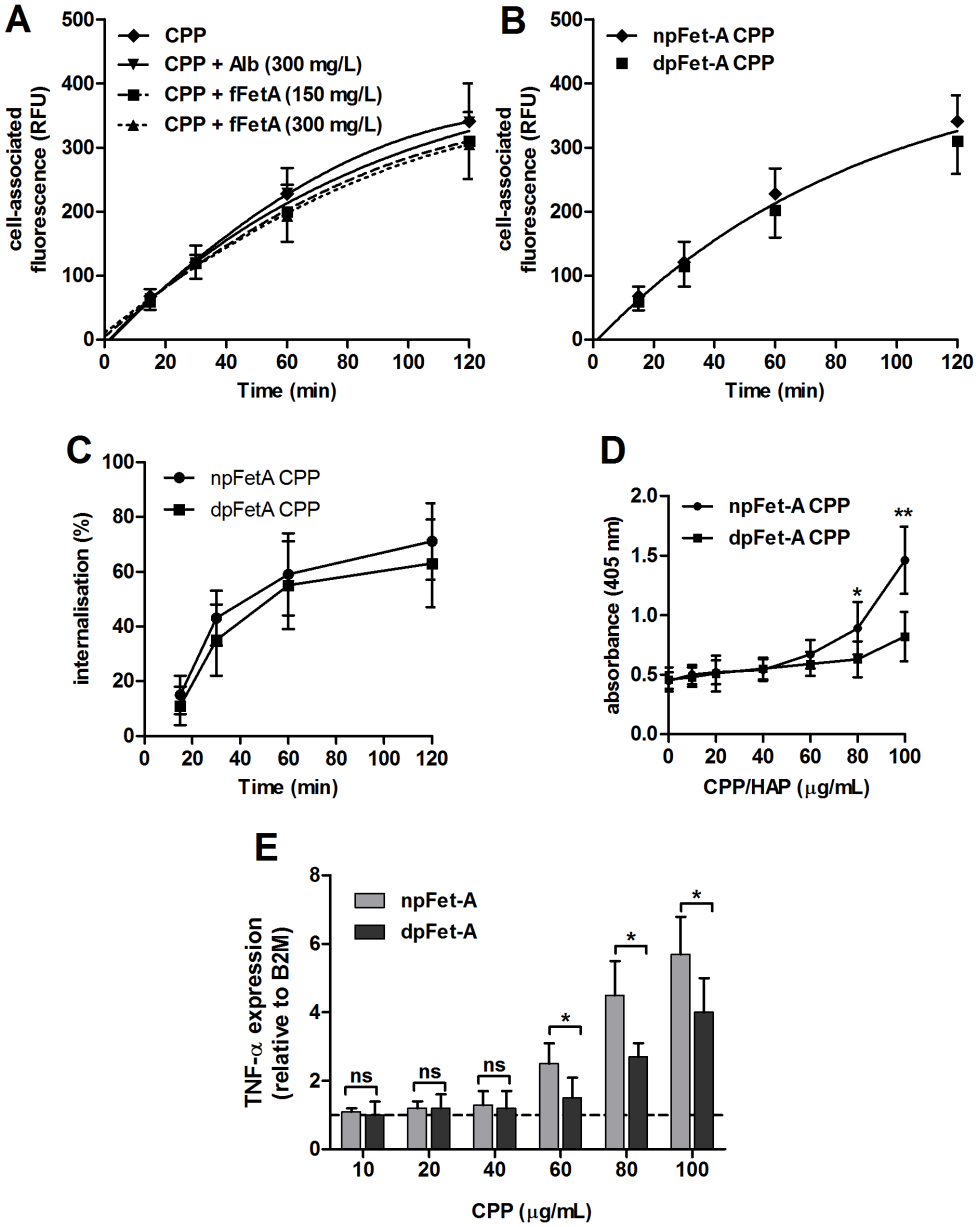
The effect of fetuin-A phosphorylation on CPP uptake, induction of TNF-α expression and apoptosis in RAW 264.7 macrophages. **A**, adherent RAW 264.7 cells were incubated for 15 to 120 minutes with AlexaFluor 488-labeled fetuin-A-containing CPP (100 µg/mL) at 37°C in the presence or absence of monomeric free fetuin-A or albumin at the concentrations indicated. Cell-associated fluorescence was measured using a microplate reader (n = 12 for each time-point). **B**, Cells incubated with CPP synthesized using either AlexaFluor 488-labeled natively phosphorylated (np) or dephosphorylated (dp) fetuin-A-containing CPP (both at 100 µg/mL), showed no significant difference in cell-associated fluorescence (n = 8 for each treatment, F-test, P = 0.149). **C**, CPP internalization was calculated as the ratio of quenched signal (intracellular CPP) to unquenched signal (combined cell-surface and intracellular CPP) after correction for the unquenchable fluorescence at each time point. Cell-surface fluorescence was quenched using anti-AlexaFluor488 IgG (80 µg/mL) or PBS as control for 1 h at 4°C. Internalization was unaffected by the presence of free fetuin-A and albumin (data not shown). **D**, Increased apoptosis of RAW 264.7 cells incubated in npFet-A-containing CPP medium compared to dpFet-A-CPP after 24 h treatment. Apoptosis was detected by measuring ssDNA levels by ELISA (n = 8 for each treatment). **E**, shows the dose-dependent effect of npFet-A or dpFet-A-containing CPP exposure on TNF-α gene expression assessed by qPCR, after 24 h incubation (P for trend both <0.01, ANOVA). mRNA levels were normalized to B2 M and expressed relative to control medium (1: dashed line). npFet-A-containing CPP induced a greater increase in TNF-α expression than dpFet-A-containing CPP at 60 µg/mL and above (n = 4 for each treatment). Determinations were made in 3 independent experiments and are expressed as mean ± SD. Pairwise comparisons were made using the unpaired *t*-test; ns, not significant (P>0.05); *P<0.05; **P<0.01.

To study the kinetics of CPP internalization, we measured the increase in cell-associated fluorescence of adherent RAW 264.7 cells cultured in 96-well plates and incubated with AlexaFluor488-labelled fetuin-A-containing CPP (100 µg/mL) (Fig. 7A). After incubation for up to 120 minutes at 37°C, cells were washed with ice-cold PBS and incubated on ice, with or without (PBS only) quenching of cell-surface fluorescence with 80 µg/mL anti-AlexaFluor488 IgG for 1 h. Unquenchable cell-surface fluorescence (∼5–15%) was estimated by incubation of cells with labeled CPP on ice before addition of quenching antibody (data not shown). Internalization of CPP was estimated by calculating the ratio of quenched fluorescence (intracellular CPP) to unquenched signal (combined cell-surface and intracellular CPP) after correction for the unquenchable fluorescence at each time point. As shown in Figure 7C, CPP internalization increased rapidly from 15 to 30 minutes and then increased steadily from 60 to 120 minutes. Pre-incubation of labeled-CPP or cells with culture media containing unlabeled ‘free’ fetuin-A (at 150 mg/L and 300 mg/L) or albumin had no significant effect on cell-associated fluorescence (Fig. 7A) or CPP internalization (F-test, all P>0.1).

### Fetuin-A Phosphorylation does not Effect CPP Uptake but Modulates the Pro-inflammatory Response

Previously we reported that phosphorylated fetuin-A was the main fetuin-A species present in CPP circulating in human serum [30]. We speculated that phosphorylation may affect cellular uptake, possibly due to differences in particle surface charge. CPP synthesized using enzymatically-dephosphorylated fetuin-A (dpFet-A), also subsequently labeled with AlexaFluor488, showed similar uptake into RAW 264.7 cells compared to natively-phosphorylated fetuin-A (npFetA)-containing CPP at the same concentration (Fig. 7B and Fig. 7C). Indeed, even after 120 minutes, uptake of dpFet-A CPP and npFet-A CPP only differed by 8%, which may be accounted for by the slightly higher degree of labeling of the npFet-A CPP preparation (7.6 vs. 7.4 mol dye/mol protein) or imprecision of the fluorometric measurements. Interestingly however, we observed a greater increase in macrophage apoptosis with npFet-A-containing CPP compared to dpFet-A CPP at high levels (Fig. 7D). dpFet-A-containing CPP were also found to induce significantly lower TNF-α expression compared to the same concentrations of npFet-A-containing CPP (Fig. 7E). Indeed, at 100 µg/mL, dpFet-A-CPP had only 68% of the stimulatory effect of npFet-A CPP on TNF-α expression.

### CPP Exposure Up-regulates Expression of the Macrophage Scavenger Receptor-A *in vitro*

**Figure 8:**
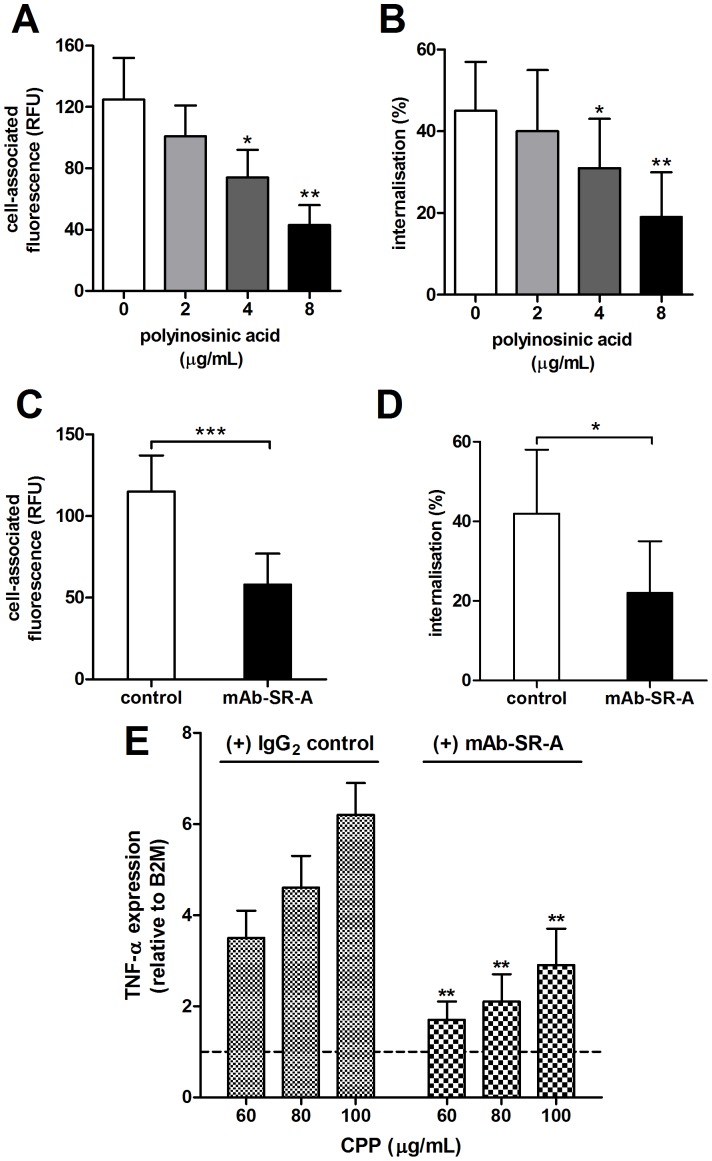
SR-AI/II pathway is a major route of CPP uptake and mediates downstream effects on TNF-α expression in RAW 264.7 macrophages. Adherent RAW 264.7 cells were pre-incubated with **A–B**, polyinosinic acid, at the concentrations indicated, or **C–D**, SR-AI/II blocking antibody (or IgG_2b_ isotype control, both at 10 µg/mL) prior to addition of Alexa Fluor 488-labeled fetuin-A-containing CPP (100 µg/mL) for a further 30 min. Cell-associated fluorescence and internalization was determined as before (n = 12 for each time-point). Both pre-treatments significantly reduced cell-associated fluorescence and CPP uptake compared to controls, consistent with SR-AI/II pathway being a major route of CPP clearance. **E**, pre-incubation of cells with SR-AI/II blocking antibody (10 µg/mL) also resulted in a significant reduction in CPP-induced TNF-α expression after 24 h treatment with CPP-containing culture medium relative to pre-treatment with IgG_2b_ isotype antisera (10 µg/mL). mRNA levels were normalized to B2 M and expressed relative to control medium (1: dashed line). Determinations were made in 3 independent experiments and are expressed as mean ± SD. Pairwise comparisons were made using the unpaired *t*-test; ns, not significant (P>0.05); *P<0.05; **P<0.01.

**Figure 9:**
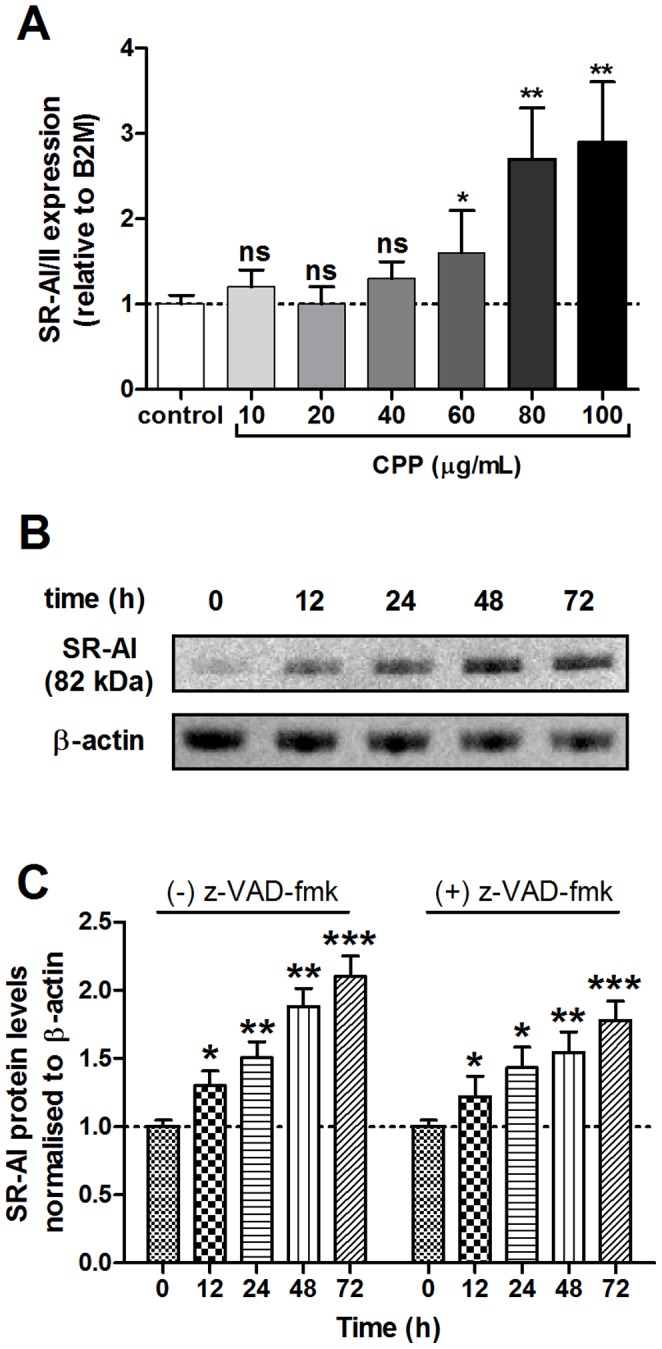
CPP induce SR-AI/II expression in RAW 264.7 macrophages. Murine RAW 264.7 cells were treated with CPP-containing culture medium for 24, 48 and 72 h, and at the concentrations indicated. **A**, shows the dose-dependent effect of CPP exposure on SR-AI/II gene expression in cells treated for 24 h (P for trend = 0.01, ANOVA). mRNA levels were normalized to B2 M and expressed relative to control medium (1: dashed line). **B**, representative Western blot of whole-cell lysate SR-AI protein levels 12 to 72 h after incubation in culture medium containing 60 µg/mL CPP. **C**, SR-AI protein levels normalized to β-actin and expressed relative to control levels, with or without pre-treatment of cells with 20 µmol/L z-VAD-fmk (pan-caspase inhibitor). All determinations were made in triplicate and in 3 independent experiments and are expressed as mean ± SD. Pairwise comparisons were made using the unpaired *t*-test; ns, not significant (P>0.05); *P<0.05; **P<0.01.

Consistent with the findings of Herrmann *et al* [23], that CPP are endocytosed by macrophage via the type I or II class A scavenger receptor (SR-AI/II), we found that pre-incubation of RAW 264.7 cells with the competitive scavenger receptor ligand, polyinosinic acid (polyI), showed a dose-dependent reduction in cell-associated fluorescence (mean RFU 125±27 vs. 43±13, P<0.001) and in CPP internalization, 30 minutes after the addition of CPP (Fig. 8A and 8B). Similarly, pre-incubation of cells with SR-AI neutralizing antibody significantly reduced cell-associated fluorescence (mean RFU 115±22 vs. 58±19, P<0.001) and CPP uptake at 30 minutes Fig. 8C and 8D). Consistent with CPP binding/uptake via the SR-AI being required for downstream effects on cytokine secretion, TNF-α-induced cytokine expression was significantly attenuated in cells treated with SR-AI blocking antibody after 24 h incubation, compared to cells pre-incubated with control IgG (Fig. 8E). Finally, we considered the effect of CPP exposure on SR-AI/II expression itself. After 24 h incubation with CPP, a significant increase in SR-AI/II expression was observed at high CPP levels (Fig. 9A: 60–100 µg/mL) and remained elevated over control levels for 72 h (data not shown). Western blotting of whole-cell lysates also revealed that SR-AI protein levels (relative to β-actin) were significantly increased within 12 h exposure to 60 µg/mL (Fig. 9B and 9C). Likewise, cells pre-incubated with z-VAD-fmk (20 µmol/L) also showed a significant increase in SR-AI protein levels after exposure to 60 µg/mL CPP for 12 h (Fig. 9C).

## Discussion

Here we have demonstrated, for the first time, that high levels of human fetuin-A-containing calciprotein particles (CPP) elicit a pro-inflammatory response from macrophage *in vitro*. Uptake of CPP by RAW 264.7 cells induced expression and release of TNF-α and IL-1β in a dose-dependent manner. CPP uptake was also found to induce cellular oxidative stress, as indicated by increased 8-iso-PGF_2α_ production, and apoptosis at very high levels. Importantly, these effects were not seen at low levels of CPP, suggesting that these potentially harmful effects are only likely to be encountered in extreme pathological states where CPP synthesis is very high, or where chronic exposure has overwhelmed clearance capacity. In support of such a role in health, we have found that circulating serum CPP are undetectable by the current, albeit fairly insensitive, and indirect methodology [30]. Thus, we speculate that low-level physiological CPP production (if any) might be protective, and unlikely to present a significant pro-inflammatory insult.

Another major finding, was that CPP markedly attenuated the effects on cytokine expression and cell viability compared to hydroxyapatite (HAP) crystals of similar size and calcium content. This builds upon the original study by Terkeltaub *et al* [20], who found that human fetuin-A specifically and strongly inhibited the stimulatory effect of serum-coated HAP crystals on superoxide release from neutrophils. Indeed, in the present study, iNOS expression and 8-iso-PGF_2α_ levels were significantly lower in CPP compared to HAP-treated cells. Synthetic CPP were also found to have a marginally greater stimulatory effect on macrophage than serum-derived CPP at very high levels. The reason for this is not clear, but may reflect the inhibitory activity of other serum components variably present in serum CPP (e.g. fibrinogen, fibronectin, haptoglobin, apolipoprotein A_1_, α_ 1_-acid glycoprotein, osteoprotegerin, bone morphogenetic protein 2) [20], [29], [30].

There is now a strong body of evidence to suggest that fetuin-A is anti-inflammatory in most contexts, and has been shown to reduce the inflammatory response to injury and a variety of toxic stimuli in a number of animal models [43]–[48]. Here we provided evidence that fetuin-A-coated mineral is less pro-inflammatory than naked calcium phosphate nanocrystals, and is consistent with the notion that calciprotein particle formation and clearance represents an important humoral defense mechanism against the potentially cytotoxic effects of nanocrystalline mineral [49].

Work in VSMC has demonstrated that cellular uptake of calcium phosphate crystals results in a rapid rise in intracellular calcium concentration (presumably as a result of crystal dissolution), leading to loss of membrane integrity and cell death [7]. Of particular relevance to the present study, Ewence *et al* found that BCP crystals pre-incubated in 1% FCS before addition to VSMC was reported to have no significant effect on intracellular calcium concentration, suggesting that the interaction of calcium phosphate crystals with serum components may attenuate their affect on cell viability [7]. The precise intracellular mechanisms involved here remain elusive, but we speculate that mineral coating by fetuin-A may affect the dissolution kinetics of the calcium phosphate core, and once internalized and acidified within the phagolyosomal pathway, may give rise to a less pronounced spike in intracellular calcium concentration. An alternative hypothesis, is that fetuin-A (either free or that liberated from the mineral core) may have a direct stabilizing effect on cell viability. With respect to the apparent enhancement of macrophage survival, one possibility is that fetuin-A may block caspase-mediated apoptotic pathways. Indeed, in cultured human aortic VSMC, fetuin-A was found to reduce apoptotic events by inhibiting cleavage of caspases 3, 8 and 9 [50]. Since we found that free fetuin-A had no significant effect on CPP uptake, a finding entirely consistent with that of Herrmann *et al* who also found that free bovine fetuin-A had no effect on synthetic CPP clearance by macrophage [23], we conclude that the positive effect of fetuin-A on cell viability appears to result from direct action within the cell. The mechanistic details, however, of how fetuin-A might ameliorate the pro-apoptoic effects of calcium phosphate crystal uptake have yet to be fully elucidated.

Haglund *et al* demonstrated that 7–20% of the fetuin-A circulating in plasma was phosphorylated, predominantly at Ser312 and to a lesser extent at Ser120 [51]. Recent mass spectrometry based studies of human phosphoproteome have identified four other serine residues that may be partially phosphorylated: Ser134, Ser138, Ser325, Ser334 [52], [53]. With respect to fetuin-A phosphorylation, we previously reported that phospho-fetuin-A was the main fetuin-A species present in serum CPP [30]. Relatedly, Matsui *et al* found only phosphorylated fetuin-A in CPP harvested from the serum of rats with adenine-induced renal failure [32]. However, the functional significance of this protein modification remains obscure, as own data [30], in addition to that of others [33], suggests that fetuin-A phosphorylation is dispensable for CPP formation and does not enhance calcification inhibitory potential. In the present study we found that fetuin-A phosphorylation had little effect on CPP uptake, but did have a significant effect on the nature of the downstream intracellular response. Indeed, CPP synthesized using fully dephosphorylated fetuin-A (dpFet-A) demonstrated only 50% of the increase in TNF-α seen with CPP made with natively-phosphorylated fetuin-A (npFet-A). This points to the possibility that fetuin-A phosphorylation may serve as a ‘signal’ within the cell. Further work is needed to explore the action, and fate, of different fetuin-A phospho-isoforms once present within the intracellular environment.

Extensive studies by Herrmann and colleagues recently identified that the type I and type II class A scavenger-receptor (SR-AI) was responsible for CPP clearance by phagocytic cells of the reticuloendothelial system in the liver and spleen [23]. These key findings are in agreement with an earlier study by Nagayama *et al*, who found that fetuin-A mediated the clearance of negatively charged nanoparticles by Kupffer cells also via scavenger receptors [54]. SR-AI/II is a 220 kDa homotrimeric type II integral membrane protein that is involved in the clearance of modified phospholipoproteins (e.g. oxidized LDL, oxLDL) and other oxidized self-antigens [55]. The SR-AI/II also plays a critical role in the removal of various pathogens, apoptotic cell debris, and in the regulation of macrophage cytokine production [55]. Intriguingly, we found that exposure of macrophages to high levels of CPP up-regulated expression and protein levels of SR-AI/II. It is possible that the apoptotic environment resulting from CPP treatment may have led to upregulated SR-AI/II expression in the remaining viable, potentially, CPP-resistant cells. However, there are two lines of evidence which suggest that CPP may also have a direct effect on SR-AI/II expression, rather than indirectly, as a response to the apoptotic milieu. Firstly, increased SR-AI/II expression was already detectable at CPP levels that did not induce significant apoptosis compared to control treatment with vehicle media alone; secondly, SR-AI protein levels were also elevated in RAW 264.7 cells pre-treated with the pan-caspase inhibitor, z-VAD-fmk, and exposed to growth media containing 60 µg/mL CPP for 24 h. Speculatively, increased calcium influx and/or ROS generation due to CPP uptake may induce a stress-response via mitogen-activated protein kinase/c-Jun N-terminal kinase pathways leading to transactivation of SR-AI/II gene expression by activator protein-1 [56], [57]. Further work is needed to clarify how CPP exposure induces SR-AI/II expression in the macrophage.

Irrespective of the exact mechanism, these findings would suggest that high levels of CPP, or chronic exposure to CPP, as typically seen in patients with CKD, would be expected to result in increased SR-AI/II expression. Indeed, it is well established that SR-AI/II expression is up*-*regulated by uraemic serum [58], [59], which if anything would be expected to result in enhanced clearance of CPP. Why then, in CKD and dialysis patients, are circulating CPP levels elevated compared to healthy controls? It may be the case that clearance pathways become saturated when chronically exposed to high levels of CPP and other competing SR-AI/II ligands. Indeed, serum concentrations of oxLDL are also generally elevated in patients with CKD [30], [60]. In fact, in our previous clinical study, serum oxLDL levels were strongly correlated with CPP levels [30]. Given the common uptake pathway it is perhaps unsurprising that the effects of excessive oxLDL and CPP on macrophage appear similar [61]; increased inflammatory cytokine production, increased nitric oxide biosynthesis, up-regulation of SR-AI/II expression and increased cell death. Finally, like oxLDL, CPP have been shown to accumulate in atherosclerotic lesions [23]. Given the findings of the present study, macrophage exposure to CPP may contribute to atherogenesis and the cytotoxic effects of atheromatous “gruel” [40].

It is however worth highlighting that SR-AI inhibition, by chemical or immunological means, did not entirely block CPP uptake or CPP-induced pro-inflammatory cytokine expression. Indeed, in the study by Herrmann *et al* [23], CPP uptake was only reduced by ∼50% in macrophage derived from SR-AI/II-deficient mice. Thus other clearance pathways may play a role in CPP metabolism, particularly in disease states and when SR-AI/II pathways become saturated.

### Clinical Significance

In the context of uraemia, elevated CPP levels may represent a response to mineral stress, but may also directly contribute to the pathogenesis of vascular calcification. Inflammatory factors like TNF-α and IL-1β have been shown to induce the de-differentiation of contractile VSMC to a synthetic mineralizing phenotype, which is considered a key event in the development of arterial calcification [62]. Chronic elevation of inflammatory cytokines has also been shown to drive skeletal osteolysis [63], potentially increasing the efflux of mineral from bone, theoretically, increasing the production of CPP. Thus the induction and elaboration of pro-inflammatory signals from macrophages that we have observed with high levels of CPP, *in vitro*, may be expected to exacerbate vascular calcification and bone demineralization. Whether failure to remove CPP from the circulation leads to the initiation or seeding of vascular mineralization, or whether CPP at levels encountered *in vivo* can potentiate these effects, has yet to be demonstrated. Animal models are now needed to translate these findings to the *in vivo* situation, and to test ways in which to harness this data therapeutically.

## References

[1] Nadra I, Mason JC, Philippidis P, Florey O, Smythe CD, et al (2005) Proinflammatory activation of macrophages by basic calcium phosphate crystals via protein kinase C and MAP kinase pathways: a vicious cycle of inflammation and arterial calcification? Circ Res 96: 1248–1256. 15905460 10.1161/01.RES.0000171451.88616.c2

[2] Jin C, Frayssinet P, Pelker R, Cwirka D, Hu B, et al (2011) NLRP3 inflammasome plays a critical role in the pathogenesis of hydroxyapatite-associated arthropathy. Proc Natl Acad Sci U S A 108: 14867–14872. 21856950 10.1073/pnas.1111101108 PMC3169126

[3] Narayan S, Pazar B, Ea HK, Kolly L, Bagnoud N, et al (2011) Octacalcium phosphate crystals induce inflammation in vivo through interleukin-1 but independent of the NLRP3 inflammasome in mice. Arthritis Rheum 63: 422–433. 21279999 10.1002/art.30147

[4] Pazar B, Ea HK, Narayan S, Kolly L, Bagnoud N, et al (2011) Basic calcium phosphate crystals induce monocyte/macrophage IL-1beta secretion through the NLRP3 inflammasome in vitro. J Immunol 186: 2495–2502. 21239716 10.4049/jimmunol.1001284

[5] Meng ZH, Hudson AP, Schumacher HR Jr, Baker JF, Baker DG (1997) Monosodium urate, hydroxyapatite, and calcium pyrophosphate crystals induce tumor necrosis factor-alpha expression in a mononuclear cell line. J Rheumatology 24: 2385–2388. 9415647

[6] Sage AP, Lu J, Tintut Y, Demer LL (2011) Hyperphosphatemia-induced nanocrystals upregulate the expression of bone morphogenetic protein-2 and osteopontin genes in mouse smooth muscle cells in vitro. Kidney Int 79: 414–422. 20944546 10.1038/ki.2010.390 PMC3198856

[7] Ewence AE, Bootman M, Roderick HL, Skepper JN, McCarthy G, et al (2008) Calcium phosphate crystals induce cell death in human vascular smooth muscle cells: a potential mechanism in atherosclerotic plaque destabilization. Circ Res 103: e28–34. 18669918 10.1161/CIRCRESAHA.108.181305

[8] Halverson PB, Greene A, Cheung HS (1998) Intracellular calcium responses to basic calcium phosphate crystals in fibroblasts. Osteoarthritis Cartilage 6: 324–329. 10197167 10.1053/joca.1998.0131

[9] McCarthy GM, Augustine JA, Baldwin AS, Christopherson PA, Cheung HS, et al (1998) Molecular mechanism of basic calcium phosphate crystal-induced activation of human fibroblasts. Role of nuclear factor kappab, activator protein 1, and protein kinase c. J Biol Chem 273: 35161–35169. 9857053 10.1074/jbc.273.52.35161

[10] Brogley MA, Cruz M, Cheung HS (1999) Basic calcium phosphate crystal induction of collagenase 1 and stromelysin expression is dependent on a p42/44 mitogen-activated protein kinase signal transduction pathway. J Cell Physiol 180: 215–224. 10395291 10.1002/(SICI)1097-4652(199908)180:2<215::AID-JCP9>3.0.CO;2-J

[11] Reuben PM, Brogley MA, Sun Y, Cheung HS (2002) Molecular mechanism of the induction of metalloproteinases 1 and 3 in human fibroblasts by basic calcium phosphate crystals. Role of calcium-dependent protein kinase C alpha. J Biol Chem 277: 15190–15198. 11836255 10.1074/jbc.M200278200

[12] Vengrenyuk Y, Carlier S, Xanthos S, Cardoso L, Ganatos P, et al (2006) A hypothesis for vulnerable plaque rupture due to stress-induced debonding around cellular microcalcifications in thin fibrous caps. Proc Natl Acad Sci U S A 103: 14678–14683. 17003118 10.1073/pnas.0606310103 PMC1595411

[13] London GM, Marchais SJ, Guerin AP, Metivier F (2005) Arteriosclerosis, vascular calcifications and cardiovascular disease in uremia. Curr Opin Nephrol Hypertens 14: 525–531. 16205470 10.1097/01.mnh.0000168336.67499.c0

[14] Morgan MP, McCarthy GM (2002) Signaling mechanisms involved in crystal-induced tissue damage. Curr Opin Rheumatol 14: 292–297. 11981329 10.1097/00002281-200205000-00017

[15] Ea HK, Liote F (2009) Advances in understanding calcium-containing crystal disease. Curr Opin Rheumatol 21: 150–157. 19339926 10.1097/BOR.0b013e3283257ba9

[16] McCarthy GM, Cheung HS (2009) Point: Hydroxyapatite crystal deposition is intimately involved in the pathogenesis and progression of human osteoarthritis. Curr Rheumatol Rep 11: 141–147. 19296887 10.1007/s11926-009-0020-6

[17] Schmid K, McSharry WO, Pameijer CH, Binette JP (1980) Chemical and physicochemical studies on the mineral deposits of the human atherosclerotic aorta. Atherosclerosis 37: 199–210. 7426095 10.1016/0021-9150(80)90005-2

[18] Nadra I, Boccaccini AR, Philippidis P, Whelan LC, McCarthy GM, et al (2008) Effect of particle size on hydroxyapatite crystal-induced tumor necrosis factor alpha secretion by macrophages. Atherosclerosis 196: 98–105. 17350022 10.1016/j.atherosclerosis.2007.02.005

[19] Laquerriere P, Grandjean-Laquerriere A, Jallot E, Balossier G, Frayssinet P, et al (2003) Importance of hydroxyapatite particles characteristics on cytokines production by human monocytes in vitro. Biomaterials 24: 2739–2747. 12711520 10.1016/s0142-9612(03)00089-9

[20] Terkeltaub RA, Santoro DA, Mandel G, Mandel N (1988) Serum and plasma inhibit neutrophil stimulation by hydroxyapatite crystals. Evidence that serum alpha 2-HS glycoprotein is a potent and specific crystal-bound inhibitor. Arthritis Rheum 31: 1081–1089. 2844196 10.1002/art.1780310901

[21] Jahnen-Dechent W, Heiss A, Schafer C, Ketteler M (2011) Fetuin-a regulation of calcified matrix metabolism. Circ Res 108: 1494–1509. 21659653 10.1161/CIRCRESAHA.110.234260

[22] Heiss A, DuChesne A, Denecke B, Grotzinger J, Yamamoto K, et al (2003) Structural basis of calcification inhibition by alpha 2-HS glycoprotein/fetuin-A. Formation of colloidal calciprotein particles. J Biol Chem 278: 13333–13341. 12556469 10.1074/jbc.M210868200

[23] Herrmann M, Schafer C, Heiss A, Graber S, Kinkeldey A, et al (2012) Clearance of fetuin-A–containing calciprotein particles is mediated by scavenger receptor-A. Circ Res 111: 575–584. 22753077 10.1161/CIRCRESAHA.111.261479

[24] Merx MW, Schafer C, Westenfeld R, Brandenburg V, Hidajat S, et al (2005) Myocardial stiffness, cardiac remodeling, and diastolic dysfunction in calcification-prone fetuin-A-deficient mice. J Am Soc Nephrol 16: 3357–3364. 16177000 10.1681/ASN.2005040365

[25] Schafer C, Heiss A, Schwarz A, Westenfeld R, Ketteler M, et al (2003) The serum protein alpha 2-Heremans-Schmid glycoprotein/fetuin-A is a systemically acting inhibitor of ectopic calcification. J Clin Invest 112: 357–366. 12897203 10.1172/JCI17202 PMC166290

[26] Westenfeld R, Schafer C, Kruger T, Haarmann C, Schurgers LJ, et al (2009) Fetuin-A protects against atherosclerotic calcification in CKD. J Am Soc Nephrol 20: 1264–1274. 19389852 10.1681/ASN.2008060572 PMC2689898

[27] Ketteler M, Bongartz P, Westenfeld R, Wildberger JE, Mahnken AH, et al (2003) Association of low fetuin-A (AHSG) concentrations in serum with cardiovascular mortality in patients on dialysis: a cross-sectional study. Lancet 361: 827–833. 12642050 10.1016/S0140-6736(03)12710-9

[28] Moe SM, Reslerova M, Ketteler M, O'Neill K, Duan D, et al (2005) Role of calcification inhibitors in the pathogenesis of vascular calcification in chronic kidney disease (CKD). Kidney Int 67: 2295–2304. 15882271 10.1111/j.1523-1755.2005.00333.x

[29] Hamano T, Matsui I, Mikami S, Tomida K, Fujii N, et al (2010) Fetuin-mineral complex reflects extraosseous calcification stress in CKD. J Am Soc Nephrol 21: 1998–2007. 20947626 10.1681/ASN.2009090944 PMC3014014

[30] Smith ER, Ford ML, Tomlinson LA, Rajkumar C, McMahon LP, et al (2012) Phosphorylated fetuin-A-containing calciprotein particles are associated with aortic stiffness and a procalcific milieu in patients with pre-dialysis CKD. Nephrology, Dialysis, Transplantation 27: 1957–1966. 10.1093/ndt/gfr609 22105144

[31] Smith ER, Cai MM, McMahon LP, Pedagogos E, Toussaint ND, et al.. (2012) Serum fetuin-A concentration and fetuin-A-containing calciprotein particles in patients with chronic inflammatory disease and renal failure. Nephrology. Dec 12. doi: 10.1111/nep.12021. [Epub ahead of print]. 10.1111/nep.12021 23231493

[32] Matsui I, Hamano T, Mikami S, Fujii N, Takabatake Y, et al (2009) Fully phosphorylated fetuin-A forms a mineral complex in the serum of rats with adenine-induced renal failure. Kidney Int 75: 915–928. 19190677 10.1038/ki.2008.700

[33] Schinke T, Amendt C, Trindl A, Poschke O, Muller-Esterl W, et al (1996) The serum protein alpha2-HS glycoprotein/fetuin inhibits apatite formation in vitro and in mineralizing calvaria cells. A possible role in mineralization and calcium homeostasis. J Biol Chem 271: 20789–20796. 8702833 10.1074/jbc.271.34.20789

[34] Gostring L, Chew MT, Orlova A, Hoiden-Guthenberg I, Wennborg A, et al (2010) Quantification of internalization of EGFR-binding Affibody molecules: Methodological aspects. Int J Oncol 36: 757–763. 20198317 10.3892/ijo_00000551

[35] Stephens AS, Stephens SR, Morrison NA (2011) Internal control genes for quantitative RT-PCR expression analysis in mouse osteoblasts, osteoclasts and macrophages. BMC Res Notes 4: 410. 21996334 10.1186/1756-0500-4-410 PMC3204251

[36] Pasch A, Farese S, Graber S, Wald J, Richtering W, et al (2012) Nanoparticle-based test measures overall propensity for calcification in serum. J Am Soc Nephrol 23: 1744–1752. 22956818 10.1681/ASN.2012030240 PMC3458464

[37] Heiss A, Jahnen-Dechent W, Endo H, Schwahn D (2007) Structural dynamics of a colloidal protein-mineral complex bestowing on calcium phosphate a high solubility in biological fluids. Biointerphases 2: 16–20. 20408632 10.1116/1.2714924

[38] Ea HK, Uzan B, Rey C, Liote F (2005) Octacalcium phosphate crystals directly stimulate expression of inducible nitric oxide synthase through p38 and JNK mitogen-activated protein kinases in articular chondrocytes. Arthritis Res Ther 7: R915–926. 16207333 10.1186/ar1763 PMC1257419

[39] Hamilton JA, McCarthy G, Whitty G (2001) Inflammatory microcrystals induce murine macrophage survival and DNA synthesis. Arthritis Res 3: 242–246. 11438042 10.1186/ar308 PMC34113

[40] Li W, Ostblom M, Xu LH, Hellsten A, Leanderson P, et al (2006) Cytocidal effects of atheromatous plaque components: the death zone revisited. FASEB J 20: 2281–2290. 17077305 10.1096/fj.06-6114com

[41] Chen X, Deng C, Tang S, Zhang M (2007) Mitochondria-dependent apoptosis induced by nanoscale hydroxyapatite in human gastric cancer SGC-7901 cells. Biol Pharm Bull 30: 128–132. 17202672 10.1248/bpb.30.128

[42] Ea HK, Monceau V, Camors E, Cohen-Solal M, Charlemagne D, et al (2008) Annexin 5 overexpression increased articular chondrocyte apoptosis induced by basic calcium phosphate crystals. Ann Rheum Dis 67: 1617–1625. 18218665 10.1136/ard.2008.087718

[43] Wang H, Zhang M, Bianchi M, Sherry B, Sama A, et al (1998) Fetuin (alpha2-HS-glycoprotein) opsonizes cationic macrophagedeactivating molecules. Proc Natl Acad Sci U S A 95: 14429–14434. 9826717 10.1073/pnas.95.24.14429 PMC24390

[44] Dziegielewska KM, Andersen NA (1998) The fetal glycoprotein, fetuin, counteracts ill-effects of the bacterial endotoxin, lipopolysaccharide, in pregnancy. Biol Neonate 74: 372–375. 9742266 10.1159/000014055

[45] Ombrellino M, Wang H, Yang H, Zhang M, Vishnubhakat J, et al (2001) Fetuin, a negative acute phase protein, attenuates TNF synthesis and the innate inflammatory response to carrageenan. Shock 15: 181–185. 11236900 10.1097/00024382-200115030-00004

[46] Wang H, Li W, Zhu S, Li J, D'Amore J, et al (2010) Peripheral administration of fetuin-A attenuates early cerebral ischemic injury in rats. J Cereb Blood Flow Metab 30: 493–504. 19953099 10.1038/jcbfm.2009.247 PMC2860738

[47] Li W, Zhu S, Li J, Huang Y, Zhou R, et al (2011) A hepatic protein, fetuin-A, occupies a protective role in lethal systemic inflammation. PLoS One 6: e16945. 21347455 10.1371/journal.pone.0016945 PMC3035675

[48] Chertov O, Ermolaeva MV, Satpaev DK, Saschenko LP, Kabanova OD, et al (1994) Inhibitory effect of calf fetuin on the cytotoxic activity of LAK cell-derived factors and tumor necrosis factor. Immunol Lett 42: 97–100. 7530233 10.1016/0165-2478(94)90042-6

[49] Herrmann M, Kinkeldey A, Jahnen-Dechent W (2012) Fetuin-a function in systemic mineral metabolism. Trends Cardiovasc Med 22: 197–201. 22902178 10.1016/j.tcm.2012.07.020

[50] Reynolds JL, Skepper JN, McNair R, Kasama T, Gupta K, et al (2005) Multifunctional roles for serum protein fetuin-a in inhibition of human vascular smooth muscle cell calcification. J Am Soc Nephrol 16: 2920–2930. 16093453 10.1681/ASN.2004100895

[51] Haglund AC, Ek B, Ek P (2001) Phosphorylation of human plasma alpha2-Heremans-Schmid glycoprotein (human fetuin) in vivo. Biochem J 357: 437–445. 11439093 10.1042/0264-6021:3570437 PMC1221970

[52] Zhou W, Ross MM, Tessitore A, Ornstein D, Vanmeter A, et al (2009) An initial characterization of the serum phosphoproteome. J Proteome Res 8: 5523–5531. 19824718 10.1021/pr900603n PMC2789176

[53] Han G, Ye M, Zhou H, Jiang X, Feng S, et al (2008) Large-scale phosphoproteome analysis of human liver tissue by enrichment and fractionation of phosphopeptides with strong anion exchange chromatography. Proteomics 8: 1346–1361. 18318008 10.1002/pmic.200700884

[54] Nagayama S, Ogawara K, Minato K, Fukuoka Y, Takakura Y, et al (2007) Fetuin mediates hepatic uptake of negatively charged nanoparticles via scavenger receptor. Int J Pharm 329: 192–198. 17005341 10.1016/j.ijpharm.2006.08.025

[55] Moore KJ, Freeman MW (2006) Scavenger receptors in atherosclerosis: beyond lipid uptake. Arterioscler Thromb Vasc Biol 26: 1702–1711. 16728653 10.1161/01.ATV.0000229218.97976.43

[56] Mietus-Snyder M, Gowri MS, Pitas RE (2000) Class A scavenger receptor up-regulation in smooth muscle cells by oxidized low density lipoprotein. Enhancement by calcium flux and concurrent cyclooxygenase-2 up-regulation. J Biol Chem 275: 17661–17670. 10837497 10.1074/jbc.275.23.17661

[57] Wu H, Moulton K, Horvai A, Parik S, Glass CK (1994) Combinatorial interactions between AP-1 and ets domain proteins contribute to the developmental regulation of the macrophage scavenger receptor gene. Mol Cell Biol 14: 2129–2139. 8114743 10.1128/mcb.14.3.2129-2139.1994 PMC358573

[58] Ando M, Gafvels M, Bergstrom J, Lindholm B, Lundkvist I (1997) Uremic serum enhances scavenger receptor expression and activity in the human monocytic cell line U937. Kidney Int 51: 785–792. 9067911 10.1038/ki.1997.110

[59] Ando M, Lundkvist I, Bergstrom J, Lindholm B (1996) Enhanced scavenger receptor expression in monocyte-macrophages in dialysis patients. Kidney Int 49: 773–780. 8648919 10.1038/ki.1996.107

[60] Samouilidou EC, Karpouza AP, Kostopoulos V, Bakirtzi T, Pantelias K, et al (2012) Lipid abnormalities and oxidized LDL in chronic kidney disease patients on hemodialysis and peritoneal dialysis. Ren Fail 34: 160–164. 22172020 10.3109/0886022X.2011.641515

[61] Greig FH, Kennedy S, Spickett CM (2012) Physiological effects of oxidized phospholipids and their cellular signaling mechanisms in inflammation. Free Radic Biol Med 52: 266–280. 22080084 10.1016/j.freeradbiomed.2011.10.481

[62] Liu Y, Shanahan CM (2011) Signalling pathways and vascular calcification. Frontiers in bioscience : a journal and virtual library 16: 1302–1314. 10.2741/3790 21196233

[63] Demer LL, Tintut Y (2008) Vascular calcification: pathobiology of a multifaceted disease. Circulation 117: 2938–2948. 18519861 10.1161/CIRCULATIONAHA.107.743161 PMC4431628

